# “Crossbreeding” NIR‐II flavchromene for PSMA‐positive prostate cancer detection and image‐guided surgery

**DOI:** 10.1002/smo.20240020

**Published:** 2024-07-04

**Authors:** Jialiang Huang, Yongkang Yao, Liao Zhang, Chenxu Yan, Zhiqian Guo

**Affiliations:** ^1^ State Key Laboratory of Bioreactor Engineering, Key Laboratory for Advanced Materials and Joint International Research Laboratory of Precision Chemistry and Molecular Engineering Feringa Nobel Prize Scientist Joint Research Center Institute of Fine Chemicals Frontiers Science Center for Materiobiology and Dynamic Chemistry School of Chemistry and Molecular Engineering East China University of Science and Technology Shanghai China

**Keywords:** fluorescence dyes, near‐infrared, NIR‐II fluorescence imaging, PSMA‐targeting

## Abstract

Prostate‐specific membrane antigen (PSMA) is known to be overexpressed in prostate cancer (PCa). The development of precise and rapid imaging technologies to monitor PSMA is crucial for early diagnosis and therapy. Fluorescence imaging in the second near‐infrared window (NIR‐II) has emerged as a powerful tool for real‐time tracking and in vivo visualization, offering high sensitivity and resolution. However, there is a lack of stable, bright and easy‐to‐implement NIR‐II fluorescent probes for PSMA targeting. Herein, we presented a PSMA‐targeting NIR‐II fluorescent probe FC‐PSMA based on π‐conjugated crossbreeding dyed strategy that affords high stability, large extinction coefficient, and good brightness. As demonstrated, FC‐PSMA displayed a high fluorescence quantum yield in fetal bovine serum (FBS). Following intravenous injection of FC‐PSMA, the tumor‐to‐normal ratio of fluorescence intensity steadily increased over time, reaching a peak at 48 h (tumor‐to‐leg ratio = 12.16 ± 0.90). This advancement enables precise identification of PC through NIR‐II fluorescence imaging, facilitating high‐performance guidance for prostate cancer resection surgery.

## Graphical abstract

Here we report a PSMA‐targeting NIR‐II fluorescent probe based on π‐conjugated crossbreeding dyed strategy with high stability, large extinction coefficient, and good brightness. As expected, FC‐PSMA exhibits precise identification of PCa through NIR‐II fluorescence imaging, providing high‐performance guidance for prostate cancer resection surgery.

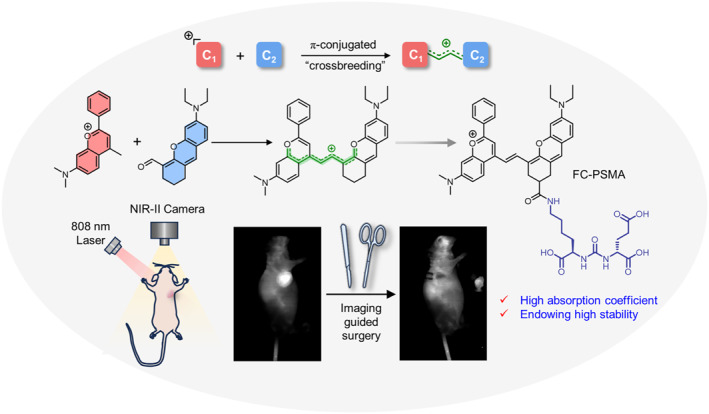

## 1 INTRODUCTION

Prostate‐specific membrane antigen (PSMA) is a type II transmembrane glycoprotein that is overexpressed on the cell membrane of both primary and metastatic prostate cancer (PCa).[^1^, ^2^, ^3^, ^4^] This characteristic has facilitated the development of various PSMA‐targeting drugs for PCa therapy.[^5^, ^6^] However, radical prostatectomy remains the most effective treatment and first choice for patients in early stages.^[7]^ Due to the irregular and indistinctive tumor boundary, excessive resection could easily damage the abundant nerves and vessels in this region, resulting in complications such as urinary incontinence and urethrostenosis.[^8^, ^9^, ^10^] Therefore, there is a high demand for the development of novel imaging technology to aid surgeons in precisely delineating tumor margins and detecting missed lesions during surgery.

**FIGURE 1:**
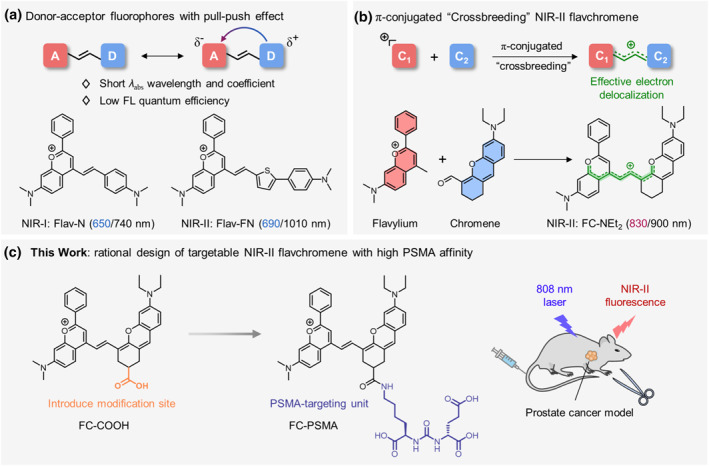
Design of π‐conjugated crossbreeding NIR‐II flavchromene with PSMA‐targeting. (a) Donor‐acceptor (d–a) fluorophores with strong push‐pull electronic effect. (b) π‐conjugated crossbreeding strategy based on Flavylium and Chromene units for constructing NIR‐II dye FC‐NEt_2_. (c) The PSMA‐targeting NIR‐II dye FC‐PSMA based on FC‐NEt_2_ scaffold for prostate cancer imaging and fluorescence‐guided surgery.

Fluorescence techniques offer a powerful approach for surgical navigation due to their rapid feedback,[^11^, ^12^, ^13^, ^14^] high sensitivity[^15^, ^16^, ^17^, ^18^] and high spatiotemporal resolution.[^19^, ^20^, ^21^, ^22^] In particular, fluorescence imaging in the second near‐infrared window (NIR‐II, 900–1700 nm) has gained significant attention for effectively reducing background interference and enabling deeper penetration with superior imaging qualities.[^23^, ^24^, ^25^, ^26^, ^27^, ^28^, ^29^, ^30^, ^31^] Serving as the foundation of NIR‐II fluorescence imaging, the development of high‐performance fluorophores has been a promising research focus for decades.[^32^, ^33^] Generally, there are two strategies for the design of NIR‐II fluorophores, one system is based on polymethine skeleton,[^34^, ^35^] and the other is a donor‐acceptor (D‐A) framework.[^36^, ^37^] Unfortunately, cyanine dyes constructed by lengthening polymethine chains suffer from poor stability (chemical stability and photo‐stability), making it challenging to incorporate targeting units into molecule skeleton.[^38^, ^39^] In comparison, D‐A type dyes rely on the push‐pull electronic effect between the donor and acceptor, exhibiting excellent stability, but short excitation wavelengths, low extinction coefficients and brightness (Figure 1a).[^40^, ^41^, ^42^] Thus, there is an urgent need to develop new molecular design strategies for constructing stable, bright and easy‐to‐implement NIR‐II fluorescent dyes for PSMA targeting.

Recently, we reported a novel π‐conjugated “crossbreeding” molecular strategy for NIR‐II fluorescent dyes.[^43^, ^44^] In contrast to typical D‐A dyes (such as Flav‐N and Flav‐FN) with a push‐pull electronic effect, π‐conjugated crossbreeding fluorophores effectively strengthened electron delocalization, and endowed little bond‐length alternation within the conjugative chain, exhibiting high stability, large extinction coefficient, good brightness and easy modification (Figure 1b). Herein, based on this π‐conjugated crossbreeding strategy, NIR‐II fluorophore flavchromene FC‐NEt_2_ was constructed, in which two chromophores (flavylium and chromene) were integrated into a single conjugative core. Then, we innovatively designed an FC‐COOH molecular scaffold that incorporated a carboxylate group as the modification site. Subsequently, (S)‐2‐(3‐((S)‐5‐amino‐1‐carboxypentyl) ureido) pentanedioic acid (Glu‐urea‐Lys)^[45]^ as a specific binding moiety towards PSMA was successfully introduced into FC‐NEt_2_ to construct the PSMA‐targeting crossbreeding NIR‐II fluorescent probe FC‐PSMA. In addition to the binding ability, the Glu‐urea‐Lys moiety with three carboxy groups also endowed FC‐PSMA with certain hydrophilicity, promoting high fluorescence quantum yield (2.52%) in FBS aqueous solution. Based on FC‐PSMA, the tumor was visualized with high resolution (tumor‐to‐leg ratio = 12.16 ± 0.90) and clear margin by NIR‐II imaging in the PCa mouse model.

## 2 RESULTS AND DISCUSSION

### 2.1 Discovering π‐conjugated crossbreeding NIR‐II flavchromene

**FIGURE 2:**
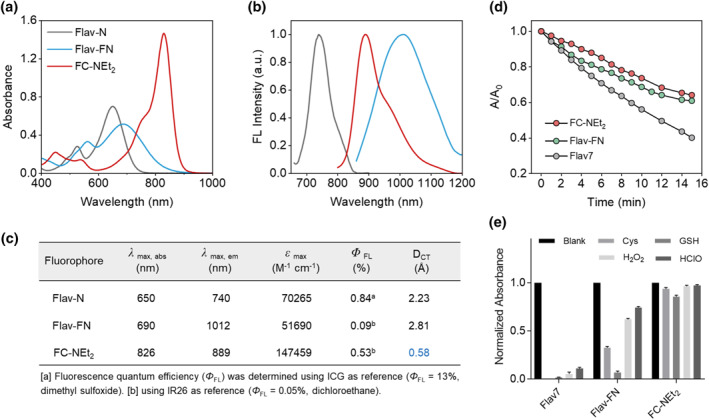
The absorption spectra (a), normalized emission spectra (b) and photophysical properties (c) of Flav‐N, Flav‐FN and FC‐NEt_2_ taken in DMSO at 10 μM (*λ*_ex_ = 650, 690 and 830 nm, respectively). (d) Photostability of NIR‐II dyes Flav‐FN, FC‐NEt_2_ and Flav7 (10 μM) in PBS/DMSO (pH 7.4, 1:1, v/v) under continuous‐wave laser exposure with a power density of 1 W cm^−2^ (690, 785, and 980 nm laser, respectively). (e) Chemical stability of Flav‐FN, FC‐NEt_2_ and Flav7 (10 μM) with various agents in PBS/DMSO (pH 7.4, 1:1, v/v), Cys: 100 μM; GSH: 1 mM; H_2_O_2_: 100 μM; HClO: 100 μM.

As shown in Figure 2, the photophysical characteristics of Flav‐N, Flav‐FN, and FC‐NEt_2_ were measured and summarized. Notably, FC‐NEt_2_ demonstrated exceptional optical properties with a maximum absorption peak at approximately 830 nm and a maximum emission peak near 900 nm in DMSO, extending up to 1200 nm (Figure 2a,b). It exhibits a high fluorescence quantum yield of 0.53%, compared to the reference quantum yield of IR26 in dichloroethane at 0.05%. Moreover, FC‐NEt_2_ displays significantly higher absorption coefficients (147,459 M^−1^ cm^−1^) than Flav‐N (70,265 M^−1^ cm^−1^) and Flav‐FN (51,690 M^−1^ cm^−1^). The calculated charge transfer distance (DCT) 0.58 Å of FC‐NEt_2_ is much lower than 2.23 Å of Flav‐N and 2.81 Å of Flav‐FN, suggesting effective electron delocalization in *π*‐conjugated crossbreeding structure of FC‐NEt_2_ (Figure 2c). To assess the photostability of NIR‐II fluorescent dyes, both Flav‐FN, FC‐NEt_2_ and Flav7 were irradiated with lasers (690, 785 and 980 nm, respectively) at a power density of 1 W cm^−2^, and their maximum absorption values were monitored over time. FC‐NEt_2_ shows superior photostability with its maximum absorption value remaining around 64% after laser irradiation for 15 min, outperforming both Flav‐FN (61%) and Flav7 (40%) as shown in Figure 2d. Subsequent tests were conducted to assess the chemical stability of the three NIR‐II fluorescent dyes against representative reactive oxygen species (HClO and H_2_O_2_) and common biorelevant nucleophiles (glutathione (GSH) and cysteine (Cys)). As depicted in Figure 2e, after incubation with different reactive molecules at 37°C for 1 h, the maximum absorption value of FC‐NEt_2_ at 830 nm exhibited no significant decrease, indicating its excellent chemical stability and resistance to the influence of reactive molecules. In contrast, the maximum absorption values of Flav‐FN and Flav7 showed a significant decline in response to reactive oxygen species. To sum up, the π‐conjugated crossbreeding dye FC‐NEt_2_ serves as an exceptional molecular scaffold in NIR‐II fluorescence imaging for versatile biomedical applications.

### 2.2 Constructing PSMA‐targeting flavchromene

**FIGURE 3:**
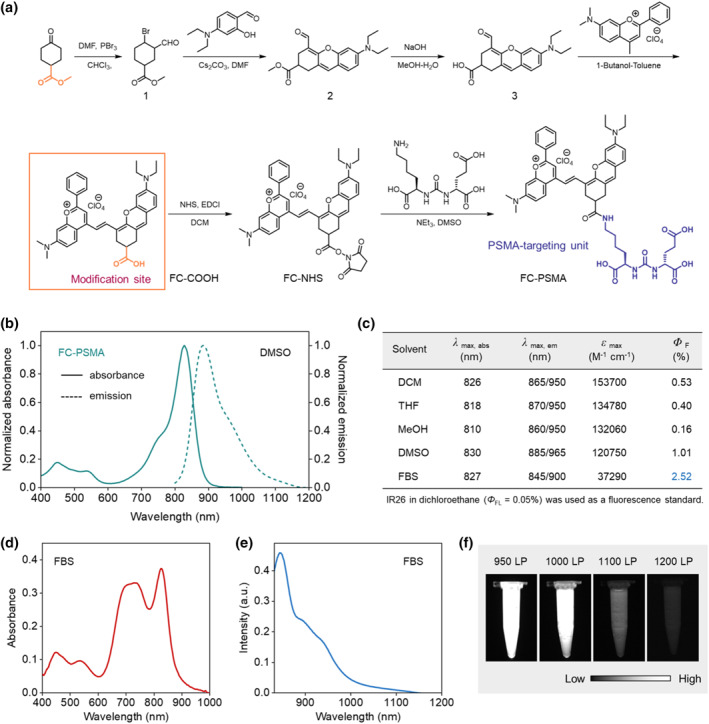
(a) Synthesis route of PSMA‐targeting NIR‐II fluorescent probe FC‐PSMA. (b) The normalized absorption and emission spectra of FC‐PSMA taken in DMSO at 10 μM, *λ*_ex_ = 808 nm. (c) Table of photophysical properties of FC‐PSMA in different solvents. The absorption (d) and emission spectra (e) of FC‐PSMA in FBS at 10 μM, *λ*_ex_ = 808 nm. (f) NIR‐II fluorescence images of FC‐PSMA in FBS with various long‐pass filters at the same exposure time, *λ*_ex_ = 808 nm.

Encouraged by the excellent photophysical properties of FC‐NEt_2_, we further constructed the probe FC‐PSMA to target prostate cancer. The synthetic route of FC‐PSMA is shown in Figure 3a. Firstly, compound 2 was realized by taking vilsmiere reaction and cyclizing with 4‐(diethylamino)salicylaldehyde in moderate yield (21.0%). The methyl ester structure was used to protect the carboxy group in the above reaction conditions and removed to provide compound 3 with a high yield (73%) before the next reaction. After hydrolysis, a Knoevenagel reaction was performed between compound 3 and Flavylium to provide FC‐COOH with a high yield (75%). Then, the carboxyl group of FC‐COOH reacted with N‐hydroxy succinimide to produce the active ester (FC‐NHS) with a high yield (85%). Finally, Glu‐urea‐Lys binding moieties were conjugated with FC‐NHS via an amide bond to give FC‐PSMA with moderate yield (53%).

FC‐PSMA exhibits optical properties similar to FC‐NEt_2_ in DMSO, with a maximum absorption at around 830 nm and a maximum emission at 885 nm (Figure 3b). It possesses a high molar extinction coefficient of 120,750 M^−1^ cm^−1^ and a fluorescence quantum yield of 1.01% (Figure 3c). In FBS (tested at 25°C), FC‐PSMA demonstrates a distinct absorption peak at 830 nm and an enhanced emission peak at 850 nm, resulting in a calculated fluorescence quantum yield of 2.52%, which is 2.5 times higher than its quantum yield in DMSO (Figure 3c–e). To further validate the NIR‐II fluorescence imaging capability of FC‐PSMA under physiological conditions, NIR‐II fluorescence images were captured using different wavelength filters (950–1200 nm) in the NIR‐II fluorescence imaging system. As shown in Figure 3f, the intensity of the NIR‐II fluorescence signal gradually diminishes as the filter wavelength increases. Considering both the intensity of the fluorescence signal and SNR, the 1000 nm long‐pass filter was chosen for subsequent NIR‐II fluorescence imaging experiments to achieve superior tumor imaging contrast.

To assess the stability of FC‐PSMA in physiological environment, the fluorescence intensity of FC‐PSMA at 1000 nm in FBS was monitored over time (Figure S2A). The results demonstrated that the fluorescence of FC‐PSMA remained almost unaffected within 6 h, indicating its suitability for long‐term imaging in physiological conditions. Additionally, the photostability and chemical stability of FC‐PSMA were evaluated using the aforementioned method, and excellent performance was observed in both aspects within FBS (Figure S2B,C). After confirming the exceptional photophysical properties and stability of the FC‐PSMA probe, its cytotoxicity was investigated. Human prostate cancer (LNCap) cells were used for testing and incubated with different concentrations of FC‐PSMA and the reference dye FC‐NEt_2_ for 24 h before conducting cell viability analysis through methyl thiazolyl tetrazolium (MTT) assay. As shown in Figure S3, even at concentrations as high as 50 μM, LNCap cell viability remained above 85% when treated with either FC‐PSMA or reference dye FC‐NEt_2_. These findings highlight the remarkable biocompatibility of FC‐PSMA, rendering it suitable for biological imaging applications in vivo.

### 2.3 Real‐time tumor‐specific NIR‐II imaging in prostate cancer

Following confirmation of low cytotoxicity associated with the use of FC‐PMSA dye, our focus shifted towards investigating its NIR‐II fluorescence imaging capabilities for prostate cancer detection in vivo. Subcutaneous LNCaP tumor‐bearing mice were selected as an experimental model. After the tail vein administration of FC‐PSMA (PBS solution, 300 μM, 200 μL), a mixture (Control Group 1) of FC‐PSMA (300 μM) and 2‐PMPA (10 mM, an effective selective Glutamate Carboxypeptidase II inhibitor to bind to PSMA)^[9]^ in PBS solution (200 μL) and FC‐NEt_2_ (Control Group 2, 300 μM in PBS solution containing 1% Tween‐80, 200 μL), whole‐body NIR‐II fluorescence imaging on mice at different time points (6, 12, 24, 36, 48 h) was acquired under 808 nm laser excitation (power density of 90 mW cm^−2^) with a 1000 nm long‐pass filter and an exposure time of 100 ms.

**FIGURE 4:**
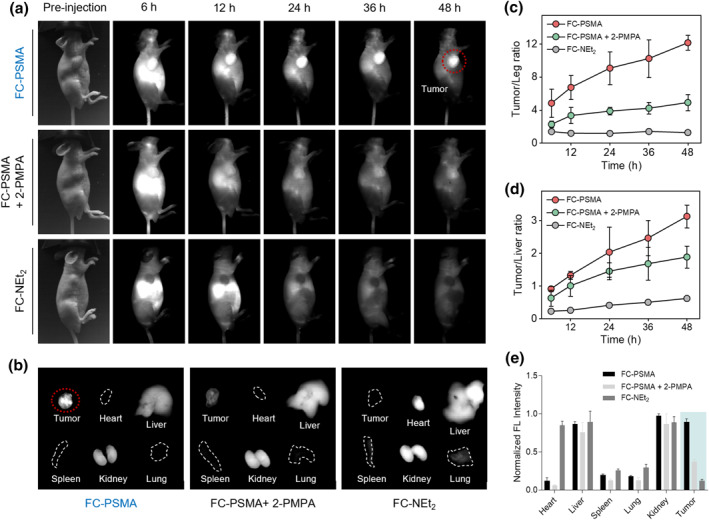
In vivo PSMA‐targeting performance of FC‐PSMA. (a) NIR‐II fluorescence imaging of LNCap tumor‐bearing mice after tail vein injection of FC‐PSMA, FC‐PSMA + 2‐PMPA and FC‐NEt_2_. (b) NIR‐II fluorescence imaging of the tumor and major organs (heart, liver, spleen, kidney and lung) after tail vein injection for 48 h. The fluorescence intensity of tumor‐to‐leg ratio (c) and tumor‐to‐liver ratio (d). (e) The normalized fluorescence intensity of major organs. 2‐PMPA is an effective selective Glutamate Carboxypeptidase II inhibitor that binds to PSMA. Laser: 808 nm; Filter: 1000 nm long‐pass; Exposure time: 100 ms.

As illustrated in Figure 4a, the tumor displayed distinct boundaries after 6 h post‐injection, and the contrast between the tumor and surrounding tissues became more pronounced over time. Notably, between 24 and 48 h post‐injection, the tumor exhibited significantly higher signal intensity compared to other regions, with a well‐defined outline. In contrast, mice co‐injected with both FC‐PSMA and 2‐PMPA showed much weaker fluorescence signals in the tumor area compared to those injected with only FC‐PSMA. Moreover, no apparent fluorescence signal was observed in the tumor region of mice injected with FC‐NEt_2_. In order to provide a more detailed demonstration of the in vivo metabolism and tumor enrichment of FC‐PSMA, quantitative analysis of fluorescence intensity was conducted on the tumor site, liver site, and leg based on the aforementioned NIR‐II fluorescence images (Figure 4c,d). The results revealed that the tumor‐to‐normal ratio (TNR), calculated as the ratio of tumor fluorescence intensity to normal tissue fluorescence intensity, continued to increase over time following injection of FC‐PSMA until peaking at 48 h (tumor/leg reaching 12.16 ± 0.90, tumor/liver at 3.13 ± 0.35). These findings indicate that FC‐PSMA exhibits remarkable targeting capabilities for prostate cancer while demonstrating excellent performance in NIR‐II in vivo fluorescence imaging.

Subsequently, at 48 h post‐injection, the mice were euthanized and tumors, along with major organs (heart, liver, spleen, lungs, and kidneys), were dissected for ex vivo NIR‐II fluorescence imaging to further investigate the biodistribution of the probe (Figure 4b,e). Consistent with the in vivo imaging results, co‐injection of FC‐PSMA and 2‐PMPA significantly attenuated the tumor fluorescence signal compared to injection of FC‐PSMA alone. In contrast, mice injected with FC‐NEt_2_ exhibited no detectable fluorescence signal in the tumors. These findings further underscore the remarkable specific targeting ability of FC‐PSMA towards prostate cancer.

### 2.4 Navigating tumor resection guided by NIR‐II fluorescence imaging

Encouraged by the remarkable specific targeting capability of the FC‐PSMA probe towards prostate cancer and the high signal‐to‐noise ratio of NIR‐II fluorescence tumor imaging, further investigation was conducted to explore the ability of FC‐PSMA in accurately guiding tumor resection during surgery using NIR‐II fluorescence imaging. The surgical procedure was performed on mice under isoflurane and oxygen gas anesthesia, and the entire process of tumor resection was recorded using an NIR‐II fluorescence live imaging system. Leveraging the outstanding photophysical properties of FC‐PSMA and its excellent brightness in vivo, a laser power density of as low as 90 mW·cm^−2^ at 808 nm was employed during the NIR‐II fluorescence imaging to ensure tissue injury did not occur. Moreover, by utilizing a 1000 nm long‐pass filter, clear images could be obtained with an exposure time of just 100 ms, enabling capture of 10 frames per second for real‐time smooth signal feedback to facilitate precise tumor resection.

Based on the previous research, tumors exhibited well‐defined outlines 24–48 h after the injection of FC‐PSMA through the tail vein. An attempt was made to perform tumor resection surgery 24 h post‐injection of FC‐PSMA (Figure S4A). Initially, an incision was made in the skin surrounding the tumor to expose the tumor mass (0–85 s), resulting in a significant increase in TNR after removing the skin from 5.29 to 7.35 (Figure S4B). Subsequently, two rounds of tumor excision were performed (85–108 s, 108–145 s), leading to a decrease in TNR from 7.35 to 2.10, indicating complete removal based on visual inspection. However, residual minute tumors could still be observed through NIR‐II fluorescence imaging (highlighted in the red box), prompting further excision (145–165 s) and resulting in a further decrease in TNR to1.97 with no remaining NIR‐II fluorescence signal detected.

**FIGURE 5:**
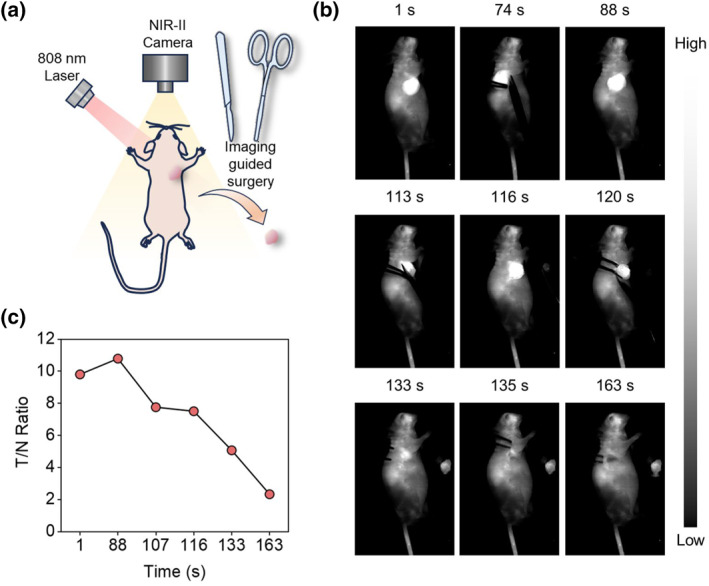
(a) Schematic illustration of NIR‐II image‐guided surgery. (b) NIR‐II fluorescence imaging‐guided tumor resection after tail vein injection of FC‐PSMA for 48 h. Laser: 808 nm; Exposure time: 100 ms; Filter: 1000 nm long‐pass. (c) Tumor‐to‐normal ratio during the surgery.

Subsequent attempts were made to perform tumor resection surgery 48 h post‐injection of FC‐PSMA (Figure 5a). Following the aforementioned steps, sequential procedures included excising the skin surrounding the tumor (1–88 s), initially (88–107 s) and secondarily (107–116 s), removing the main tumor mass, subsequently removing the main tumor mass for a third time (116–133 s), and excising the residual macroscopically invisible tumors (133–163 s). This resulted in a decrease in TNR from 9.79 to 2.23 (Figure 5b). Notably, when performing tumor resection surgery at 48 h post‐injection of FC‐PSMA, clearer boundaries of the tumors were observed. Overall, NIR‐II fluorescence imaging during surgical procedures effectively delineated tumor boundaries and facilitated the identification of minute unresected tumors, highlighting its potential for clinical translation.

## 3 CONCLUSION

In summary, we focused on developing a π‐conjugated crossbreeding NIR‐II fluorophore, FC‐NEt_2_, by integrating two chromophores (flavylium and chromene). FC‐NEt_2_ possessed enhanced electron delocalization, resulting in high chemical stability and photostability, large extinction coefficient (>10^5^ M^−1^ cm^−1^), and good brightness in the NIR‐II region. By incorporating the Glu‐urea‐Lys unit as a specific PSMA binding moiety into the FC‐NEt_2_ molecule skeleton, the obtained FC‐PSMA probe exhibited a high fluorescence quantum yield (2.52%) in FBS and precise targeting recognition of prostate cancer. Following intravenous injection of FC‐PSMA for 48 h, the TNR tumor/leg ratio reached 12.16 ± 0.90, providing high‐performance NIR‐II fluorescence imaging guidance for prostate cancer resection surgery. Overall, FC‐PSMA displayed excellent performance in visualizing prostate tumor, demonstrating great potential for clinical translation in prostate tumor resection.

## CONFLICT OF INTEREST STATEMENT

The authors declare no conflicts of interest.

## ETHICS STATEMENT

All animal experiments were performed according to the guidelines of the Care and Use of Laboratory Animals formulated by the Ministry of Science and Technology of China and were approved by the Animal Care and Use Committee of East China University of Science and Technology (2021‐07001).

## Supporting information

Supporting Information S1

Video S1

## Data Availability

The data that support the findings of this study are available from the corresponding author upon reasonable request.

## References

[1] M. J. Roberts , T. Maurer , M. Perera , M. Eiber , T. A. Hope , P. Ost , S. Siva , M. S. Hofman , D. G. Murphy , L. Emmett , W. P. Fendler , Nat. Rev. Urol. 2023, 20, 23. 36473945 10.1038/s41585-022-00670-6

[2] G. Capasso , A. Stefanucci , A. Tolomeo , Eur. J. Med. Chem. 2024, 263, 115966. 37992520 10.1016/j.ejmech.2023.115966

[3] M. Sekhoacha , K. Riet , P. Motloung , L. Gumenku , A. Adegoke , S. Mashele , Molecules 2022, 27, 5730. 36080493 10.3390/molecules27175730 PMC9457814

[4] R. J. Rebello , C. Oing , K. E. Knudsen , S. Loeb , D. C. Johnson , R. E. Reiter , S. Gillessen , T. Van der Kwast , R. G. Bristow , Nat. Rev. Dis. Primers 2021, 7, 9. 33542230 10.1038/s41572-020-00243-0

[5] A. Fenner , Nat. Rev. Urol. 2020, 17, 132. 10.1038/s41585-020-0292-1 32047282

[6] M. E. Rodnick , C. Sollert , D. Stark , M. Clark , A. Katsifis , B. G. Hockley , D. C. Parr , J. Frigell , B. D. Henderson , L. Bruton , S. Preshlock , M. Abghari‐Gerst , M. R. Piert , M. J. Fulham , S. Eberl , K. Gagnon , P. J. H. Scott , Nat. Protoc. 2022, 17, 980. 35246649 10.1038/s41596-021-00662-7

[7] F. Falkenbach , T. Maurer , Nat. Rev. Urol. 2023, 20, 704. 37648788 10.1038/s41585-023-00817-z

[8] X. Wang , Y. Chen , Y. Xiong , L. Zhang , B. Wang , Y. Liu , M. Cui , J. Med. Chem. 2023, 66, 6889. 37161996 10.1021/acs.jmedchem.3c00309

[9] L. Zhang , X. Shi , Y. Li , X. Duan , Z. Zhang , H. Fu , X. Yang , J. Tian , Z. Hu , M. Cui , J. Med. Chem. 2021, 64, 7735. 34047189 10.1021/acs.jmedchem.1c00444

[10] A. J. Costello , Nat. Rev. Urol. 2020, 17, 177. 32086498 10.1038/s41585-020-0287-y

[11] L. Huang , W. Su , L. Zhu , J. Li , W. Quan , J. Yoon , W. Lin , Angew. Chem., Int. Ed. 2023, 62, e202312632. 10.1002/anie.202217508 36578174

[12] T. Liu , X. Xia , R. Wang , X. Rong , Z. Su , J. Du , J. Fan , X. Peng , W. Sun , Adv. Funct. Mater. 2023, 33, 2304347.

[13] X. Wu , R. Wang , S. Qi , N. Kwon , J. Han , H. Kim , H. Li , F. Yu , J. Yoon , Angew. Chem., Int. Ed. 2021, 60, 15418. 10.1002/anie.202101190 33942436

[14] H. Kim , Y. R. Lee , H. Jeong , J. Lee , X. Wu , H. Li , J. Yoon , Smart Mol. 2023, 1, e20220010. 10.1002/smo.20220010 PMC12118215 40625653

[15] Y. Wu , L.‐L. Sun , H.‐H. Han , X.‐P. He , W. Cao , T. D. James , Chem. Sci. 2024, 15, 757. 38179535 10.1039/d3sc05010f PMC10762965

[16] H. Ma , Y. Lu , Z. Huang , S. Long , J. Cao , Z. Zhang , X. Zhou , C. Shi , W. Sun , J. Du , J. Fan , X. Peng , J. Am. Chem. Soc. 2022, 144, 3477. 35076232 10.1021/jacs.1c11886

[17] G. Q. Zhang , W. Feng , Z. Gao , G. L. Zhang , X. Wu , Y. Xiao , X. Li , L. Zheng , D. Ding , J. Guo , B. Situ , Aggregate 2022, 4, e286.

[18] S. He , P. Cheng , K. Pu , Nat. Biomed. Eng. 2023, 7, 281. 36941352 10.1038/s41551-023-01009-1

[19] S. Zeng , Y. Wang , C. Chen , H. Kim , X. Liu , M. Jiang , Y. Yu , Y. S. Kafuti , Q. Chen , J. Wang , X. Peng , H. Li , J. Yoon , Angew. Chem., Int. Ed. 2024, 63, e202316487. 10.1002/anie.202316487 38197735

[20] J. Liu , W. Zhang , X. Wang , Q. Ding , C. Wu , W. Zhang , L. Wu , T. D. James , P. Li , B. Tang , J. Am. Chem. Soc. 2023, 145, 19662. 37655757 10.1021/jacs.3c04303 PMC10510312

[21] Y. Hu , J. Yu , M. Xu , K. Pu , J. Am. Chem. Soc. 2024, 146, 12656. 10.1021/jacs.4c02070 38683724

[22] Y. Yao , P. Ding , C. Yan , Y. Tao , B. Peng , W. Liu , J. Wang , M. A. Cohen Stuart , Z. Guo , Angew. Chem., Int. Ed. 2023, 62, e202218983. 10.1002/anie.202218983 36700414

[23] F. Wang , Y. Zhong , O. Bruns , Y. Liang , H. Dai , Nat. Photonics 2024, 18, 535. 10.1038/s41566-024-01391-5

[24] E. L. Schmidt , Z. Ou , E. Ximendes , H. Cui , C. H. C. Keck , D. Jaque , G. Hong , Nat. Rev. Methods Primers 2024, 4, 23.

[25] T. Li , Y. Zhang , F. Wu , G. Chen , C. Li , Q. Wang , Small Methods 2024, 2400132. 10.1002/smtd.202400132 38470209

[26] X. Hu , Z. Fang , C. Zhu , Y. Yang , Z. Yang , W. Huang , Adv. Funct. Mater. 2024, 2401325.

[27] F. Ren , F. Wang , A. Baghdasaryan , Y. Li , H. Liu , R. Hsu , C. Wang , J. Li , Y. Zhong , F. Salazar , C. Xu , Y. Jiang , Z. Ma , G. Zhu , X. Zhao , K. K. Wong , R. Willis , K. Christopher Garcia , A. Wu , E. Mellins , H. Dai , Nat. Biomed. Eng. 10.1038/s41551-023-01083-5 PMC11250370 37620621

[28] Z. Qin , T. B. Ren , H. Zhou , X. Zhang , L. He , Z. Li , X. B. Zhang , L. Yuan , Angew. Chem., Int. Ed. 2022, 61, e202201541. 10.1002/anie.202201541 35218130

[29] O. S. Oliinyk , C. Ma , S. Pletnev , M. Baloban , C. Taboada , H. Sheng , J. Yao , V. V. Verkhusha , Nat. Methods 2022, 20, 70. 36456785 10.1038/s41592-022-01683-0 PMC10725253

[30] J. Mu , M. Xiao , Y. Shi , X. Geng , H. Li , Y. Yin , X. Chen , Angew. Chem., Int. Ed. 2022, 61, e202114722. 10.1002/anie.202114722 34873810

[31] Q. Xu , Y. Zhang , M. Zhu , C. Yan , W. Mao , W.‐H. Zhu , Z. Guo , Chem. Sci. 2023, 14, 4091. 37063795 10.1039/d3sc00193h PMC10094356

[32] Y. Zeng , J. Qu , G. Wu , Y. Zhao , J. Hao , Y. Dong , Z. Li , J. Shi , J. S. Francisco , X. Zheng , J. Am. Chem. Soc. 2024, 146, 9888. 38546165 10.1021/jacs.3c14805

[33] R. Wei , Y. Dong , X. Wang , J. Li , Z. Lei , Z. Hu , J. Chen , H. Sun , H. Chen , X. Luo , X. Qian , Y. Yang , J. Am. Chem. Soc. 2023, 145, 12013. 37216464 10.1021/jacs.3c00594

[34] E. D. Cosco , A. L. Spearman , S. Ramakrishnan , J. G. P. Lingg , M. Saccomano , M. Pengshung , B. A. Arús , K. C. Y. Wong , S. Glasl , V. Ntziachristos , M. Warmer , R. R. McLaughlin , O. T. Bruns , E. M. Sletten , Nat. Chem. 2020, 12, 1123. 33077925 10.1038/s41557-020-00554-5 PMC7680456

[35] X. Chen , J. Li , S. Roy , Z. Ullah , J. Gu , H. Huang , C. Yu , X. Wang , H. Wang , Y. Zhang , B. Guo , Adv. Healthcare Mater. 2024, 2304506. 10.1002/adhm.202304506 38441392

[36] H. Shen , F. Sun , X. Zhu , J. Zhang , X. Ou , J. Zhang , C. Xu , H. H. Y. Sung , I. D. Williams , S. Chen , R. T. K. Kwok , J. W. Y. Lam , J. Sun , F. Zhang , B. Z. Tang , J. Am. Chem. Soc. 2022, 144, 15391. 35948438 10.1021/jacs.2c07443

[37] K. W. Lee , Y. Gao , W. C. Wei , J. H. Tan , Y. Wan , Z. Feng , Y. Zhang , Y. Liu , X. Zheng , C. Cao , H. Chen , P. Wang , S. Li , K. T. Wong , C. S. Lee , Adv. Mater. 2023, 35, 2211632. 10.1002/adma.202211632 36868183

[38] Y. Zhang , Y. Jia , S. Zhu , SmartMat 2023, e1245.

[39] A. Martin , P. Rivera‐Fuentes , Nat. Chem. 2023, 16, 28. 38012391 10.1038/s41557-023-01367-y PMC10774129

[40] L. Wang , N. Li , W. Wang , A. Mei , J. Shao , W. Wang , X. Dong , ACS Nano 2024, 18, 4683. 38295152 10.1021/acsnano.3c12316

[41] A. Ji , H. Lou , C. Qu , W. Lu , Y. Hao , J. Li , Y. Wu , T. Chang , H. Chen , Z. Cheng , Nat. Commun. 2022, 13, 3815. 35780137 10.1038/s41467-022-31521-y PMC9250501

[42] H. Xu , D. Kim , Y. Y. Zhao , C. Kim , G. Song , Q. Hu , H. Kang , J. Yoon , Adv. Mater. 2024, 2402806. 10.1002/adma.202402806 38552256

[43] L. Zhang , Y. Zhang , W. Chi , C. Yan , Z. Zhao , X. Liu , W.‐H. Zhu , Z. Guo , ACS Mater. Lett. 2022, 4, 1493.

[44] L. Zhang , C. Yan , Y. Zhang , D. Ma , J. Huang , Z. Zhao , Y. Tao , C. Liu , J. Li , W.‐H. Zhu , Z. Guo , Chem. Commun. 2023, 59, 8388. 10.1039/d3cc01742g 37305995

[45] X. Duan , F. Liu , H. Kwon , Y. Byun , I. Minn , X. Cai , J. Zhang , M. G. Pomper , Z. Yang , Z. Xi , X. Yang , J. Med. Chem. 2020, 63, 3563. 32207938 10.1021/acs.jmedchem.9b02031

