# Supplementary material for: “Crossbreeding” NIR‐II flavchromene for PSMA‐positive prostate cancer detection and image‐guided surgery
Source: Smart Mol. 2024 Jul 4;2(3):e20240020. doi: 10.1002/smo.20240020 (PMC12118234; doi:10.1002/smo.20240020)
Supplement: Supplementary file 1 — Supporting Information S1 [file SMO2-2-e20240020-s001.docx]

Supporting Information
©Wiley-VCH 2019
69451 Weinheim, Germany

**“Crossbreeding” NIR-II flavchromene for PSMA-positive prostate cancer detection and image-guided surgery**

Jialiang Huang, Yongkang Yao, Liao Zhang, Chenxu Yan, and Zhiqian Guo*

J. Huang, Y. Yao, L. Zhang, Dr. C. Yan, Prof. Dr. Z. Guo
Department Key Laboratory for Advanced Materials and Joint International Research Laboratory of Precision Chemistry and Molecular Engineering
Feringa Nobel Prize Scientist Joint Research Center, Institute of Fine Chemicals
Frontiers Science Center for Materiobiology and Dynamic Chemistry
School of Chemistry and Molecular Engineering
East China University of Science and Technology, Shanghai 200237, China
E-mail: [guozq@ecust.edu.cn](mailto:guozq@ecust.edu.cn)

**Contents**

| **1.** | **Experimental section** | **S3-S8** |  |
| --- | --- | --- | --- |
| **2.** | **Photophysical properties** | **S8-S10** |  |
| **3.** | **Stability of FC-PSMA** | **S10** |  |
| **4.** | **Cytotoxicity of FC-NEt_2_ and FC-PSMA** | **S11** |  |
| **5.** | **NIR-II fluorescence imaging of FC-NEt_2_** | **S11** |  |
| **6.** | | **Imaging and resection prostate tumor and in vivo** | **S12** |
| **7.** | **Characterization of intermediate compounds FC-NEt_2_ and FC-PSMA** | **S13-S21** |  |
| **8.** | **Reference** | **S22** |  |

1. **Experimental section**

**Materials and general methods**

Unless special stated, all solvents and chemicals were purchased from commercial suppliers in analytical grade and used without further purification. The ^1^H and ^13^C NMR spectra were recorded on a Bruker AM 400 spectrometer, using TMS as an internal standard. High resolution mass (HRMS) spectrometry data were obtained with a Waters LCT Premier XE spectrometer. Absorption spectra were collected on a Varian Cary 500 spectrophotometer, and fluorescence spectra measurements were performed on a PTI-QM4 steady-stead fluorimeter with an InGaAs photodetector. In vivo NIR-II fluorescence images were measured with NIR-II in vivo imaging system MARS (ARTEMIS INTELLIGENT IMAGING, China).

**Calculations of fluorescence quantum yield**

Optical matching solutions of IR26 (*Φ*_fl_ = 0.05% in dichloroethane) was used as the standard to measure fluorescence quantum yield. The calculation equation is as follows:

*Φ*_s_ = *Φ*_r_(A_r_F_s_/A_s_F_r_)(n_s_^2^/n_r_^2^)

Where, r and s represent reference and sample, respectively. *n* is the refractive index of the solvent, *F* is relative integrated fluorescence intensity and *A* is the absorbance.

**In vitro cytotoxicity assay**

The cell lines were purchased from the Institute of Cell Biology (Shanghai, China). Cells were all propagated in T-75 flasks cultured at 37 ℃ under a humidified 5% CO_2_ atmosphere in RPMI-1640 medium or DMEM medium (GIBCO/Invitrogen, Camarillo, CA, USA), which were supplemented with 10 % fetal bovine serum (FBS, Biological Industry, Kibbutz Beit Haemek, Israel) and 1% penicillin-streptomycin (10,000 U mL^-1^ penicillin and 10 mg/mL streptomycin, Solarbio life science, Beijing China).

The cell cytotoxicity of FC-NEt_2_ and FC-PSMA to LNCap cells were measured by 3-(4,5-dimethylthiazol-2-yl)-2,5-diphenyltetrazolium bromide (MTT) assay. The cytotoxicity was evaluated by Cell Counting Kit-8 (Dojindo, Tokyo, Japan) according to the factory’s instruction. Cells were plated in 96-well plates in 0.1 mL volume of DMEM or RPMI-1640 medium with 10 % FBS, at a density of 1×10^4^ cells/well and added with desired concentrations of FC-NEt_2_ and FC-PSMA. After incubation for 24 h, absorbance was measured at 490 nm with a Tecan GENios Pro multifunction reader (Tecan Group Ltd., Maennedorf, Switzerland). Each concentration was measured in triplicate and used in three independent experiments. The relative cell viability was calculated by the equation: cell viability (%) = (OD_treated_/OD_control_) × 100%.

**Animal models of prostate cancer**

All animal studies were conducted with the approval of the Animal Care and Use Committee in accordance with the guidelines for the care and use of Laboratory Animals. BALB/c female nude mice aged 5-6 weeks were purchased from Shanghai Slac Laboratory Animal Co. Ltd, and kepted under standard conditions. Number of qualitative qualification: No. 20170005045288. Production Permit No.: SCXK (Shanghai) 2017-0005. BALB/c nude mice were subcutaneously injected with 10^6^ LNCap cells on the right side. When the tumors reached a diameter of approximately 8 mm, tumor-bearing mice were intravenously injected with a PBS solution of FC-PSMA (300 µM, 200 µL) via the tail vein. NIR-II fluorescence imaging of the mice was performed at different time points after the injection of FC-PSMA. Prior to imaging, the mice were anesthetized with 2.5% isoflurane. NIR-II fluorescence imaging was conducted using the NIR-II in vivo imaging system MARS. After the vivo imaging, all three groups of mice were sacrificed to dissect and image the tumor and organs including heart, spleen, kidney and lung. NIR-II fluorescence imaging was conducted with a NIR-II in vivo imaging system MARS. Filter: 1000 nm long-pass; Exposure time: 500 ms; Laser: 808 nm.

**Quantum chemical calculation details**

The density functional theory (DFT) and time-department DFT (TD-DFT) calculations were employed to understand the structural and electronic properties of these dyes using *Gaussian* 16 A.^1^ Geometries of these dyes were optimized at B3LYP/Def2-SVP level in the ground state.^2^ We confirmed that we obtained stable structures via frequency analysis. The vertical excitation properties were investigated at CAM-B3LYP/Def2-SVP level. The solvent effect (in dichloromethane) was included in all calculations using the SMD mode.^3^ The electron-hole analysis was carried out using *Multiwfn* 3.7.^4^


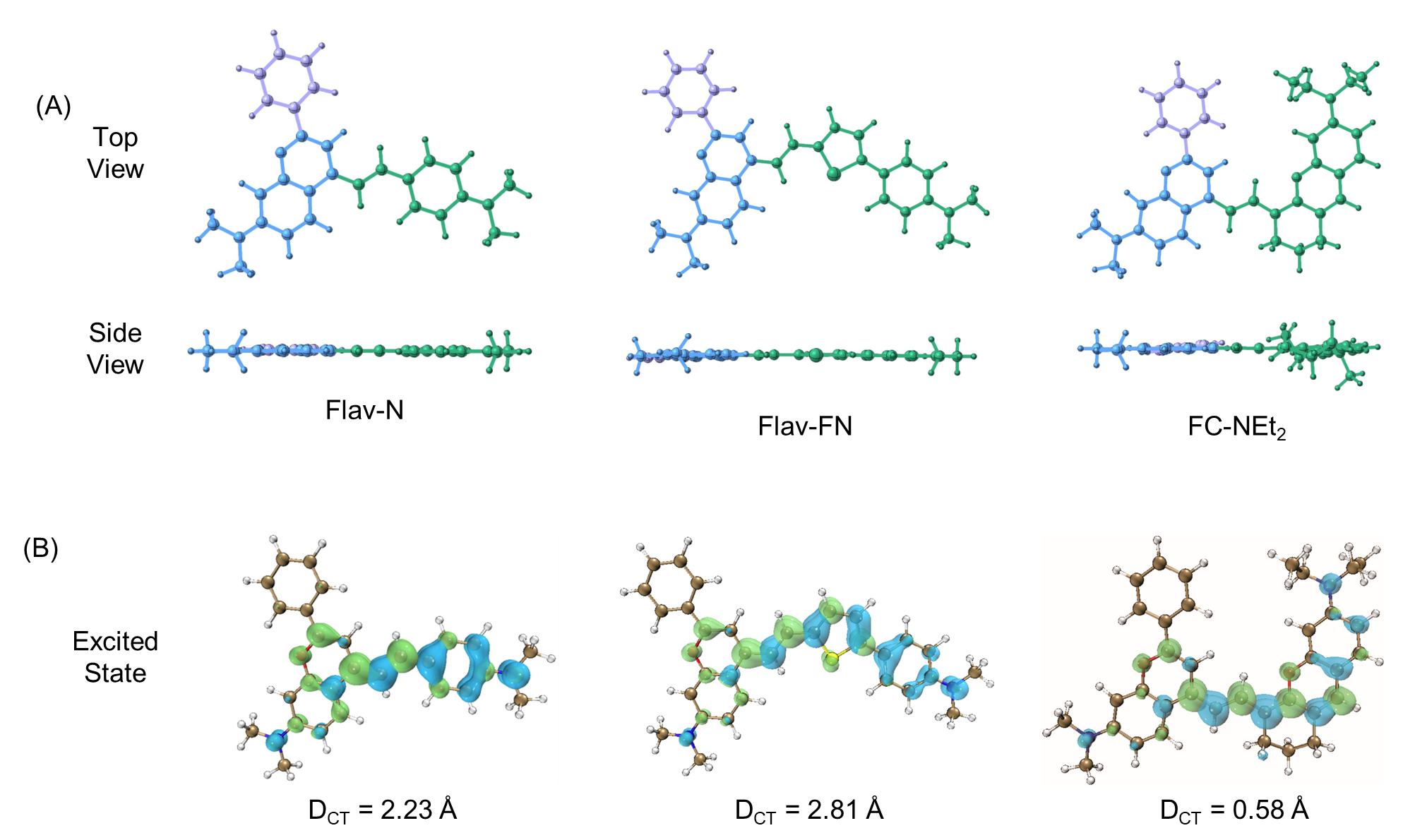


**Figure S1.** Optimized geometries (including both top and side views), the electron (green) and hole (blue) distributions and DCTs in the excited state of Flav-N, Flav-FN, and FC-NEt_2_.

**Synthesis of Flav-N, Flav-FN, FC-NEt_2_ and FC-PSMA**

The Flavylium^5^ , Chromene^6^, Flav-N^7^, Flav-FN^8^ and FC-NEt_2_^8^ was synthesized by the established procedures.


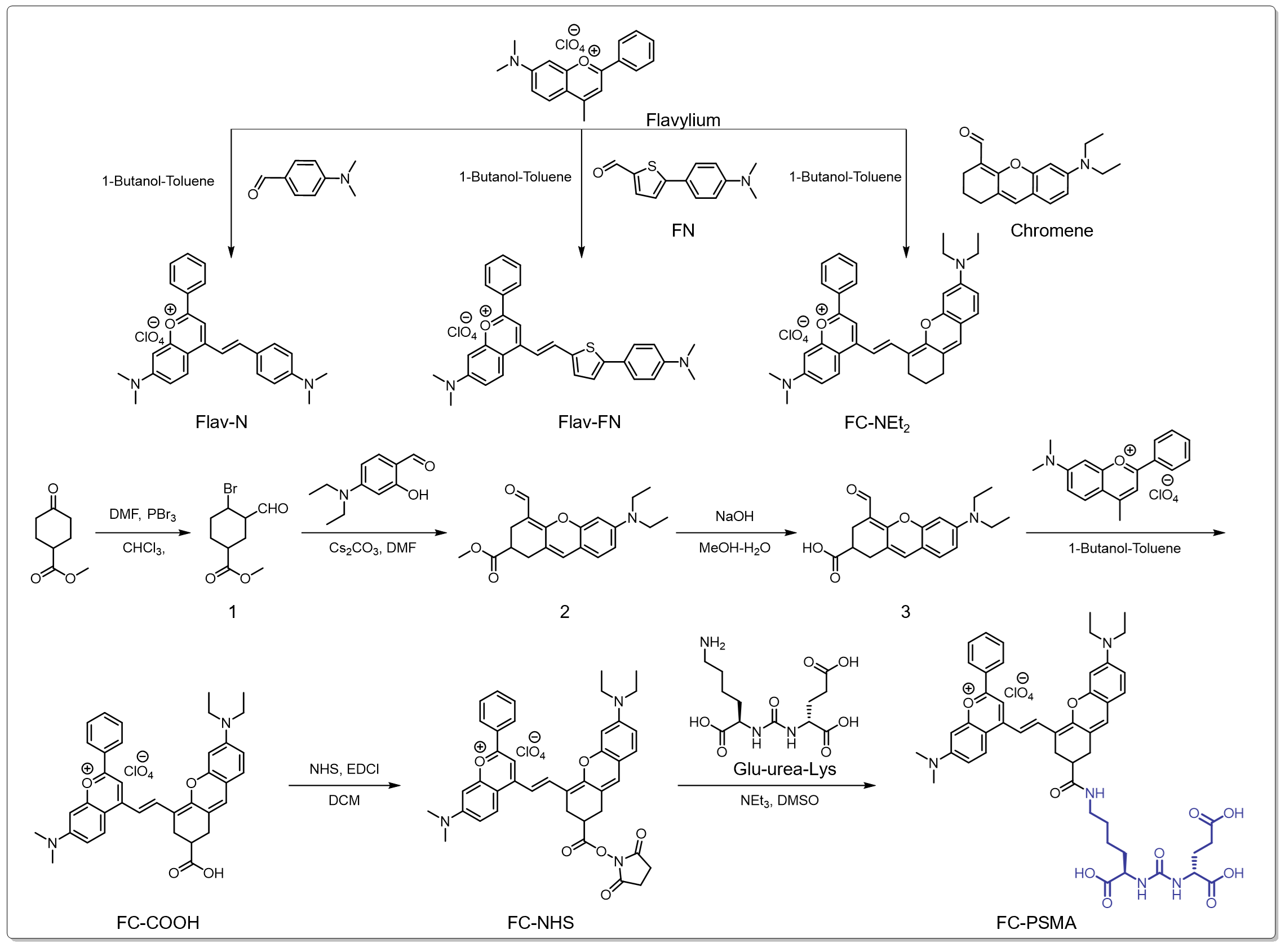


**Scheme S1**. Synthetic route of Flav-N, Flav-FN, FC-NEt_2_ and FC-PSMA

**Synthesis of FC-NEt_2_**

Flavylium (40 mg, 0.137 mmol) and Chromene (50 mg, 0.137 mmol) were dissolved in 1-Butanol (3.5 mL) and toluene (2.0 mL). Then the mixture was stirred at 120 ℃ for 10 h under an argon atmosphere. After cooling to room temperature, the mixture was filtered and the filter cake was washed with a small amount of DCM. Then the crude product was purified by silica gel chromatography using dichloromethane/methyl alcohol (v/v, 98 : 2) as the eluent to afford FC-NEt_2_ as a furvous solid (40 mg): Yield 47%. ^1^H NMR (400 MHz, DMSO-*d_6_*, ppm): *δ* 1.25 (t, *J* = 6.8 Hz, 6H, -N(CH_2_C**H**_3_)_2_), 1.84 (d, 2H, -CH_2_-), 2.73-2.81 (m, 4H, -CH_2_-), 3.16 (s, 6H, -N(CH_3_)_2_), 3.58 (q, *J* = 6.8 Hz, 4H, -N(C**H**_2_CH_3_)_2_), 6.86 (d, 1H, *J* = 2.4 Hz, Ph-H), 6.98-7.02 (m, 3H, Ph-H), 7.15 (s, 1H, Ph-H), 7.52-7.62 (m, 4H, Ph-H), 7.73 (s, 1H, Ph-H), 7.97 (s, 1H, Ph-H), 8.20 (d, *J* = 9.2 Hz, 1H, Ph-H), 8.31 (d, 2H, Ph-H), 8.69 (d , *J* = 13.6 Hz, 1H, alkene-H). Mass spectrometry (ESI positive ion mode for [M - ClO_4_]^+^): calcd for C_36_H_37_N_2_O_2_^+^ : 529.2850; found: 529.2835.

**Synthesis of** **compound 1**

To solution of DMF (2.7 mL, 38.4 mmol) and CHCl_3_ (30 mL) were added PBr_3_ (3 mL, 32.0 mmol) and stirred for 45 min at 0 ℃. After that, methyl 4-oxocyclohexanecarboxylate (2.0 g, 12.8 mmol) was added and the mixture was stirred for 24 h at room temperature. After completion of the reaction, the resultant mixture was poured into ice water and then NaHCO_3_ was added slowly until pH ≈ 7. The mixture was poured into dichloromethane and washed three times with brine. The organic layer was then separated and dried over anhydrous Na_2_SO_4_ and evaporated under reduced pressure, and **compound 1** was obtained.

**Synthesis of compound 2**

To **Compound 1** (3.2 g, 12.8 mmol) in DMF (40 mL) were added 4-(diethylamino)salicylaldehyde (2.5 g, 12.8 mmol) and Cs_2_CO_3_ (8.3 g, 25.6 mmol). Then the mixture was stirred for 24 h at 25 ℃ under an argon atmosphere. The mixture was filtered and the filter cake was washed with DCM. The organic layer was then washed with H_2_O, dried over Na_2_SO_4_, filtered and concentrated under vacuum, and then the crude product was purified by silica gel chromatography using dichloromethane/methyl alcohol (v/v, 99 : 1) as the eluent to afford **compound 2** as an orange solid (900 mg): Yield 21%.^1^H-NMR (400 Hz, DMSO-*d_6_*, ppm): *δ* 1.09-1.13 (t, *J* = 7.0 Hz, 6H, -N(CH_2_C**H**_3_)_2_), 2.30-2.36 (m, 1H, -CHCOO-), 2.64-2.84 (m, 4H, -CH_2_-), 3.38-3.44 (m, 4H, -N(C**H**_2_CH_3_)_2_), 3.63 (s, 3H, -OCH_3_), 6.53-6.55 (m, 2H, Ph-H), 7.01 (s, 1H, Ph-H), 7.16-7.18 (m, 1H, Ph-H), 10.18 (s, 1H, -CHO-H). ^13^C-NMR (100 Hz, DMSO-*d_6_*, ppm): *δ* 12.39, 23.79, 30.75, 36.23, 43.86, 51.67, 96.60, 108.07, 108.12, 109.25, 119.92, 128.13, 129.51, 149.59, 153.51, 160.47, 174.00, 185.21. Mass spectrometry (ESI positive ion mode for [M + H]^+^): calcd for C_20_H_24_NO_4_^+^ : 342.1700; found: 342.1704.

**Synthesis of compound 3**

To **Compound 2** (100 mg, 0.293 mmol) in methyl alcohol (3mL) were added 2 mL of sodium hydroxide solution (2 M). Then the mixture was stirred for 4 h at 25 ℃ under an argon atmosphere. After completion of the reaction, the resultant mixture was acidified with HCl (1 M) to a pH of 3. The mixture was extracted with DCM, and the combined organic layers were washed with H_2_O and dried over anhydrous Na_2_SO_4_. Then the crude product was purified by silica gel chromatography using dichloromethane/methyl alcohol (v/v, 97 : 3) as the eluent to afford **compound 3** as an orange solid (70 mg): Yield 73%. ^1^H-NMR (400 Hz, DMSO-*d_6_*, ppm): *δ* 1.09-1.13 (t, *J* = 7.0 Hz, 6H, -N(CH_2_C**H**_3_)_2_), 2.30-2.36 (m, 1H, -CHCOO-), 2.56-2.81 (m, 4H, -CH_2_-), 3.36-3.42 (m, 4H, -N(C**H**_2_CH_3_)_2_), 6.53-6.55 (m, 2H, Ph-H), 7.00 (s, 1H, Ph-H), 7.16-7.18 (m, 1H, Ph-H), 10.19 (s, 1H, -CHO-H). ^13^C-NMR (100 Hz, DMSO-*d_6_*, ppm): *δ* 12.39, 23.85, 30.90, 36.30, 43.86, 96.60, 108.03, 108.41, 109.30, 120.31, 128.08, 129.33, 149.54, 153.50, 160.53, 175.20, 185.28. Mass spectrometry (ESI negative ion mode for [M - H]^-^): calcd for C_19_H_20_NO_4_^-^ : 326.1398; found: 326.1391.

**Synthesis of FC-COOH**

Flavylium (100 mg, 0.275 mmol) and **compound 3** (100 mg, 0.302 mmol) were dissolved in 1-Butanol (3.5 mL) and toluene (2.0 mL). Then the mixture was stirred at 120 ℃ for 10 h under an argon atmosphere. After cooling to room temperature, the mixture was filtered and the filter cake was washed with a small amount of DCM. Then the crude product was purified by silica gel chromatography using dichloromethane/methyl alcohol (v/v, 96 : 4) as the eluent to afford **FC-COOH** as a furvous solid (170 mg): Yield 75%. ^1^H-NMR (400 Hz, DMSO-*d_6_*, ppm): *δ* 1.23-1.26 (t, *J* = 7.0 Hz, 6H, -N(CH_2_C**H**_3_)_2_), 2.79-3.10 (m, 5H, -CH_2_HCCH_2_-), 3.16 (s, 6H, N(CH_3_)_2_), 3.36-3.42 (m, 4H, -N(C**H**_2_CH_3_)_2_), 6.86-6.87 (d, *J* = 2.5 Hz, 1H, Ph-H), 6.96-7.04 (m, 3H, Ph-H), 7.10 (s, 1H, Ph-H), 7.51-7.53 (d, *J* = 9.1 Hz, 1H, Ph-H), 7.55-7.63 (m, 3H, Ph-H), 7.76 (s, 1H, Ph-H), 7.99 (s, 1H, Ph-H), 8.24-8.27 (d, *J* = 9.6 Hz, 1H, Ph-H), 8.29-8.31 (m, 2H, Ph-H), 8.66-8.70 (d, *J* = 13.8 Hz, 1H, alkene-H). ^13^C-NMR (100 Hz, DMSO-*d_6_*, ppm): *δ* 13.01, 22.56, 30.84, 37.66, 45.03, 55.39, 96.58, 97.44, 101.74, 110.45, 111.17, 112.90, 113.11, 113.74, 115.91, 122.26, 126.48, 129.25, 129.86, 131.24, 131.87, 138.87, 139.31, 146.61, 152.45, 154.25, 155.51, 156.18, 156.43, 161.46, 176.01. Mass spectrometry (ESI positive ion mode for [M - ClO_4_]^+^): calcd for C_37_H_37_N_2_O_4_^+^ : 573.2748; found: 573.2750.

**Synthesis of FC-NHS**

**FC-COOH** (100 mg, 0.149 mmol) and *N*-hydroxysuccinimide (NHS, 26 mg, 0.223 mmol) were dissolved in dichloromethane (5 mL), and then 1-(3-dimethylaminopropyl)-3-ethylcarbodiimide (hydrochloride) (EDCl, 43 mg, 0.223 mmol) was added into the solution. The system was stirred at room temperature for 6 h under argon protection. After reaction was over, the mixture was extracted with DCM, and the combined organic layers were washed with H_2_O, dried over anhydrous Na_2_SO_4_, filtered and concentrated under vacuum, and then the crude product was purified by silica gel chromatography using dichloromethane/methanol (v/v, 98 : 2) as the eluent to afford **FC-NHS** as a furvous solid (98 mg): Yield 85%. ^1^H-NMR (400 Hz, DMSO-*d_6_*, ppm): *δ* 1.23-1.27 (t, *J* = 7.0 Hz, 6H, -N(CH_2_C**H**_3_)_2_), 2.81 (s, 4H, -CH_2_CO-), 2.99-3.16 (m, 11H), 3.54-3.59 (m, 4H, -N(C**H**_2_CH_3_)_2_), 6.87-6.88 (d, *J* = 2.5 Hz, 1H, Ph-H), 6.95-7.06 (m, 3H, Ph-H), 7.10 (s, 1H, Ph-H), 7.50-7.52 (d, *J* = 9.0 Hz, 1H, Ph-H), 7.55-7.64 (m, 3H, Ph-H), 7.79 (s, 1H, Ph-H), 8.01 (s, 1H, Ph-H), 8.24-8.26 (d, *J* = 9.5 Hz, 1H, Ph-H), 8.30-8.32 (m, 2H, Ph-H), 8.68-8.72 (d, *J* = 13.8 Hz, 1H, alkene-H). ^13^C-NMR (100 Hz, DMSO-*d_6_*, ppm): *δ* 12.99, 25.93, 30.52, 35.02, 45.12, 55.39, 96.93, 97.72, 102.18, 110.56, 111.42, 112.96, 113.46, 113.55, 113.58, 113.79, 120.37, 126.65, 126.90, 129.35, 130.06, 131.34, 132.19, 139.06, 140.03, 147.66, 152.72, 154.72, 155.94, 156.37, 157.17, 160.95, 170.10, 170.56. Mass spectrometry (ESI positive ion mode for [M - ClO_4_]+): calcd for C_41_H_40_N_3_O_6_^+^ : 670.2912; found: 670.2918.

**Synthesis of FC-PSMA**

To **FC-NHS** (30 mg, 0.039 mmol) in DMSO (5 mL) were added Glu-urea-Lys (15 mg, 0.047 mmol) and triethylamine (36 μL, 0.390 mmol). Then the mixture was stirred for 24 h at 25 ℃ under an argon atmosphere. After reaction was over, the mixture washed with ethyl acetate, methylene chloride, acetonitrile and water, filtered and concentrated under vacuum to afford **FC-PSMA** as a furvous solid (20 mg): Yield 53%. ^1^H-NMR (400 Hz, DMSO-*d_6_*, ppm): *δ* 1.24-1.27 (t, *J* = 7.2 Hz, 6H, -N(CH_2_C**H**_3_)_2_), 1.34-1.38 (m, 2H, -CH_2_-), 1.48 (s, 3H, -CH_2_-), 1.67-1.74 (s, 2H, -CH_2_-), 1.91-1.95 (m, 1H, -CH_2_-), 2.22-2.27 (m, 2H, -CH_2_-), 2.64-2.68 (m, 2H, -CH_2_-), 2.81-2.88 (m, 2H, -CH_2_-), 3.17 (s, 9H, -N(CH_3_)_2_&-(CH_2_)_2_CHCOO-), 3.56-3.59 (m, 4H, -N(C**H**_2_CH_3_)_2_), 4.08-4.12 (m, 2H, -CHNCONCH-), 6.32-6.36 (t, 2H, *J* = 8.8 Hz, -NHCONH-), 6.90 (s, 1H, Ph-H), 6.98-7.15 (m, 4H, Ph-H), 7.52-7.63 (m, 4H, Ph-H), 7.76 (s, 1H, Ph-H), 8.01-8.08 (d, 2H, Ph-H&-CONH-), 8.27-8.34 (m, 3H, Ph-H), 8.71-8.75 (d, *J* = 13.2 Hz, 1H, alkene-H). Mass spectrometry (ESI positive ion mode for [M - ClO_4_]^+^): calcd for C_49_H_56_N_5_O_10_^+^ : 874.4022; found: 874.4025.

**2. Photophysical properties**


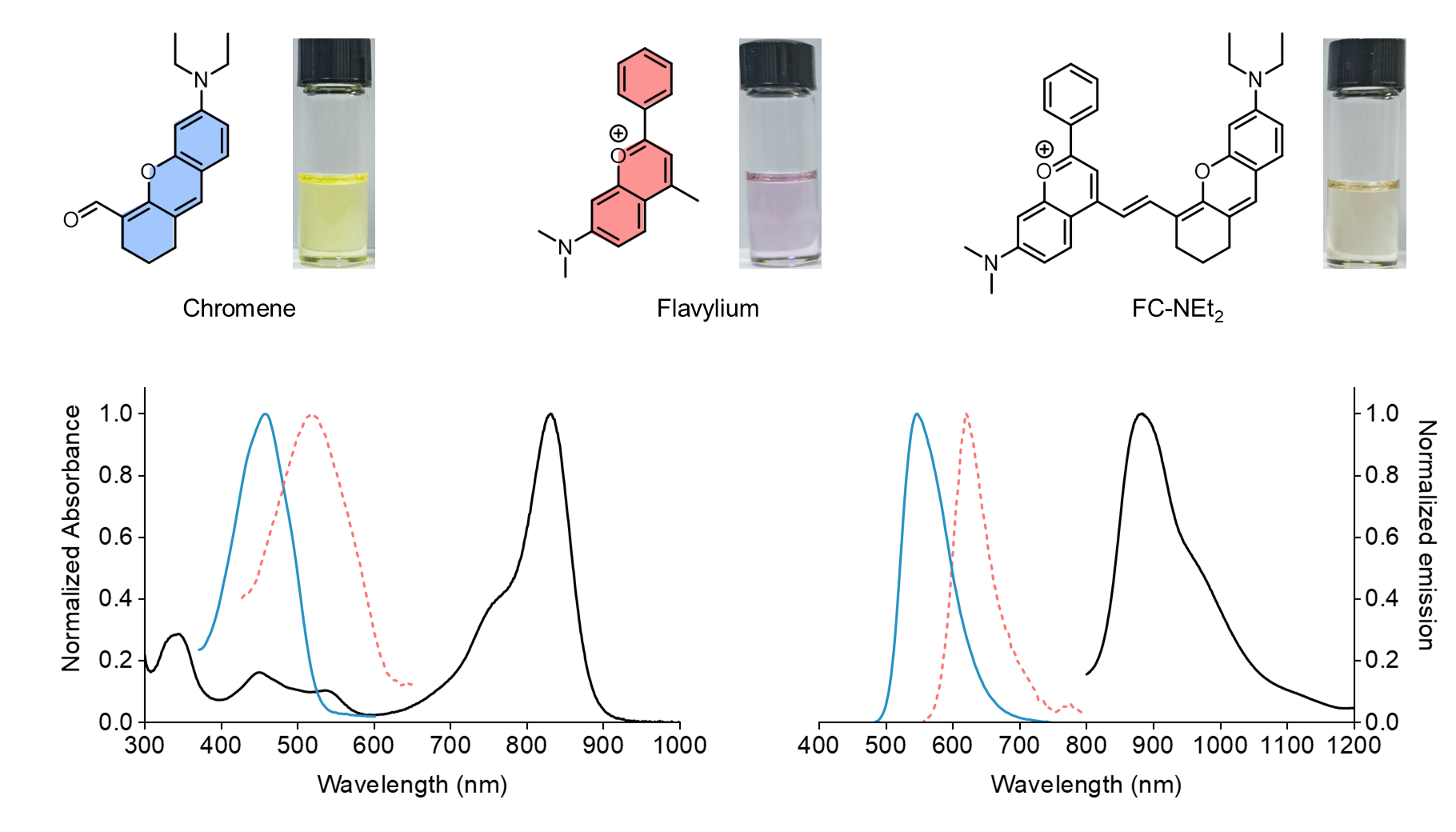


**Figure S2.** Normalized absorbance and emission spectra of chromene (λ_ex_ = 450 nm), flavylium (λ_ex_ = 520 nm) and FC-NEt_2_ (λ_ex_ = 808 nm) taken in DMSO.


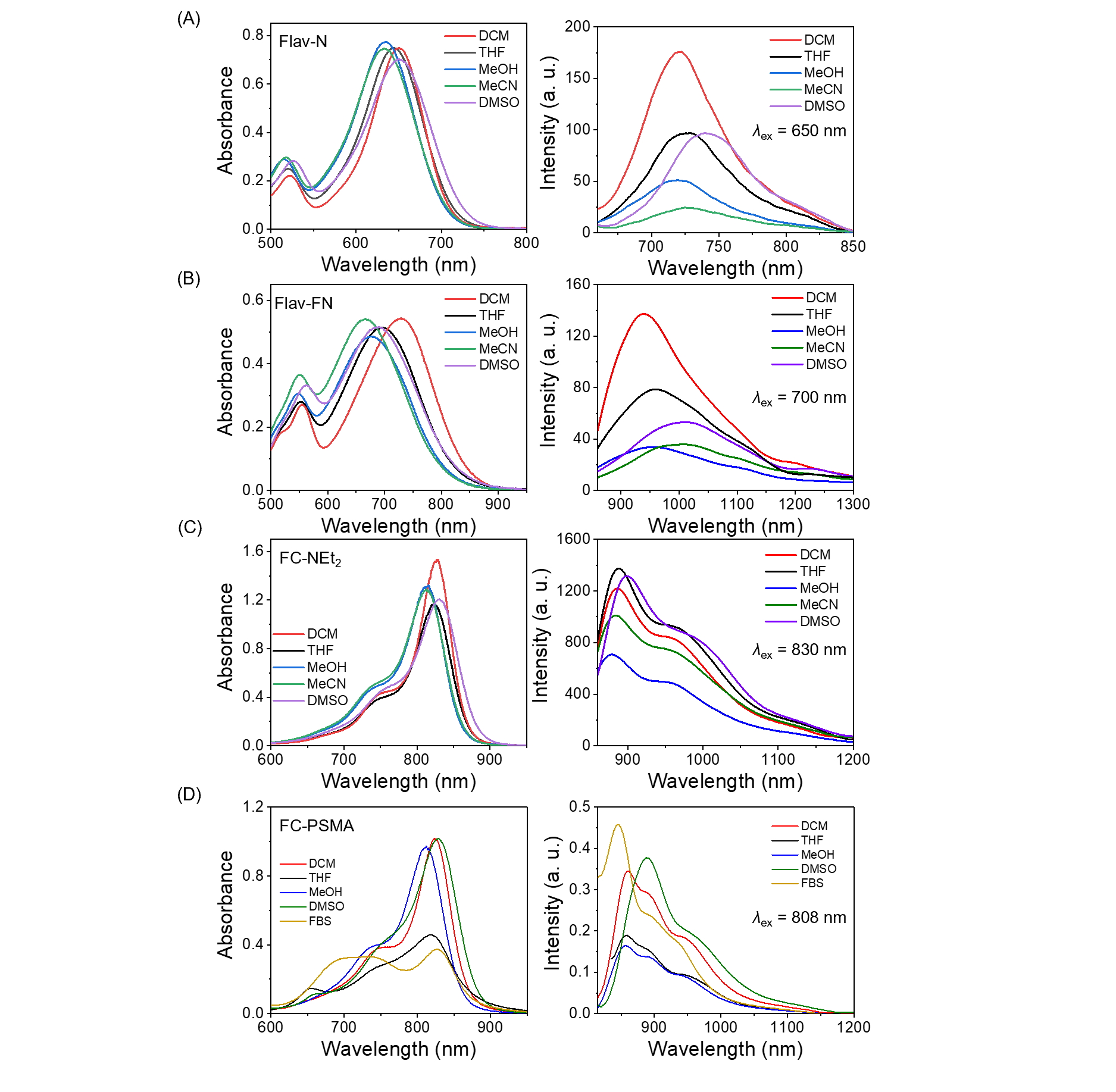


**Figure S3.** The absorption and emission spectra of Flav-N (A), Flav-FN (B), FC-NEt_2_ (C) and FC-PSMA (D) in various solvents at 25 ℃.

**Table S1.** Photophysical properties of Flav-N, Flav-FN, FC-NEt_2_ and FC-PSMA

|  | Solvent | *λ* _max, abs_  (nm) | *ε _max_*  (M^-1^ cm^-1^) | *λ* _max, em_  (nm) | *Φ* _F_  (%) |
| --- | --- | --- | --- | --- | --- |
|  | DCM | 650 | 74805 | 722 | 1.18^a^ |
|  | THF | 644 | 74936 | 728 | 0.70 ^a^ |
| Flav-N | MeOH | 634 | 77429 | 719 | 0.34 ^a^ |
|  | MeCN | 633 | 74692 | 725 | 0.19 ^a^ |
|  | DMSO | 650 | 70265 | 740 | 0.84 ^a^ |
|  | DCM | 728 | 54395 | 940 | 0.16^b^ |
|  | THF | 696 | 51400 | 960 | 0.10 ^b^ |
| Flav-FN | MeOH | 676 | 48661 | 953 | 0.04 ^b^ |
|  | MeCN | 667 | 54194 | 1009 | 0.06 ^b^ |
|  | DMSO | 691 | 51690 | 1012 | 0.09 ^b^ |
|  | DCM | 827 | 153419 | 886 | 0.40 ^b^ |
|  | THF | 822 | 116526 | 888 | 0.60 ^b^ |
| FC-NEt_2_ | MeOH | 816 | 132052 | 881 | 0.20 ^b^ |
|  | MeCN | 813 | 129078 | 883 | 0.49 ^b^ |
|  | DMSO | 831 | 120814 | 899 | 0.53 ^b^ |

(a)Fluorescence quantum efficiency (*Φ* _F_) was determined using ICG as reference *Φ* _F_ = 13%, DMSO). [b] using IR26 as reference (*Φ* _F_ = 0.05%, dichloroethane).

**3. Stability of FC-PSMA**


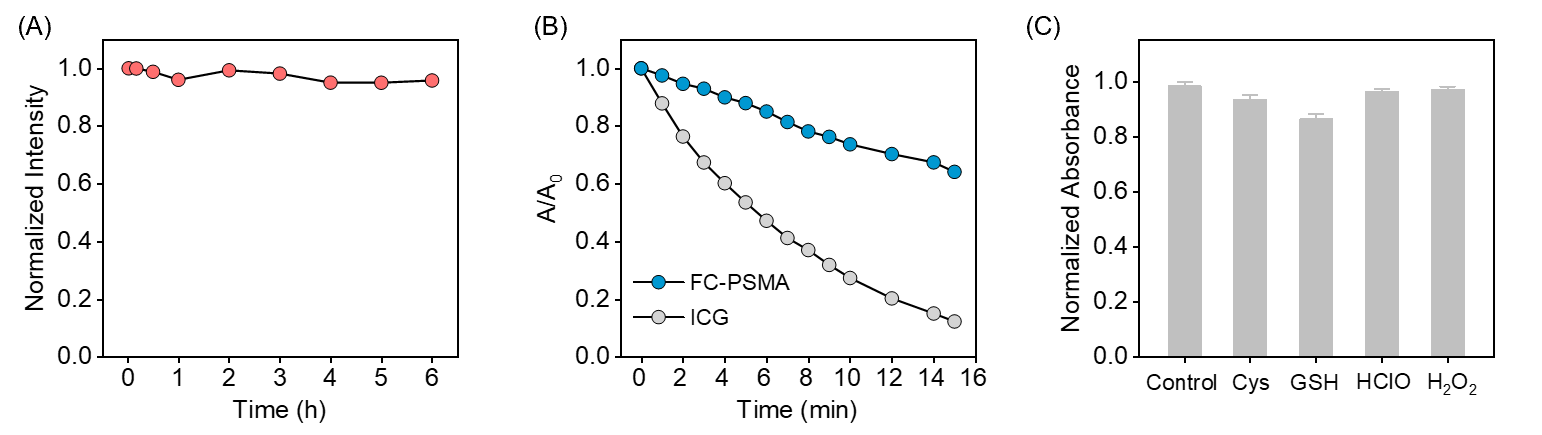


**Figure S4.** (A) Time-dependent fluorescence intensity at 1000 nm of FC-PSMA (10 μM) in FBS, λ_ex_ = 808 nm; (B) Photostability of FC-PSMA (10 μM) under continuous-wave laser exposure (808 nm, 1 W·cm^-2^) in FBS; (C) Chemical stability of FC-PSMA (10 μM) in PBS with various agents. Cys: 100 μM; GSH: 1 mM; H_2_O_2_: 100 μM; HClO: 100 μM.

**4. Cytotoxicity of FC-NEt_2_ and FC-PSMA**


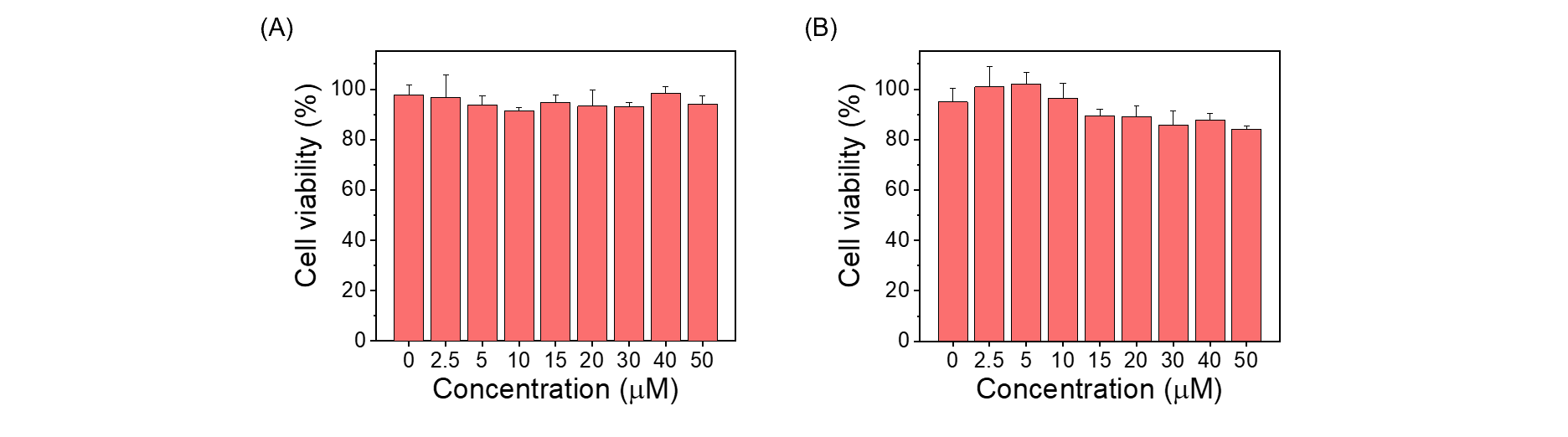


**Figure S5.** Relative viability of LNCap cells in vitro after incubation for 24 h with FC-NEt_2_ (A) and FC-PSMA (B) at various concentrations.

**5. NIR-II fluorescence imaging of FC-NEt_2_**


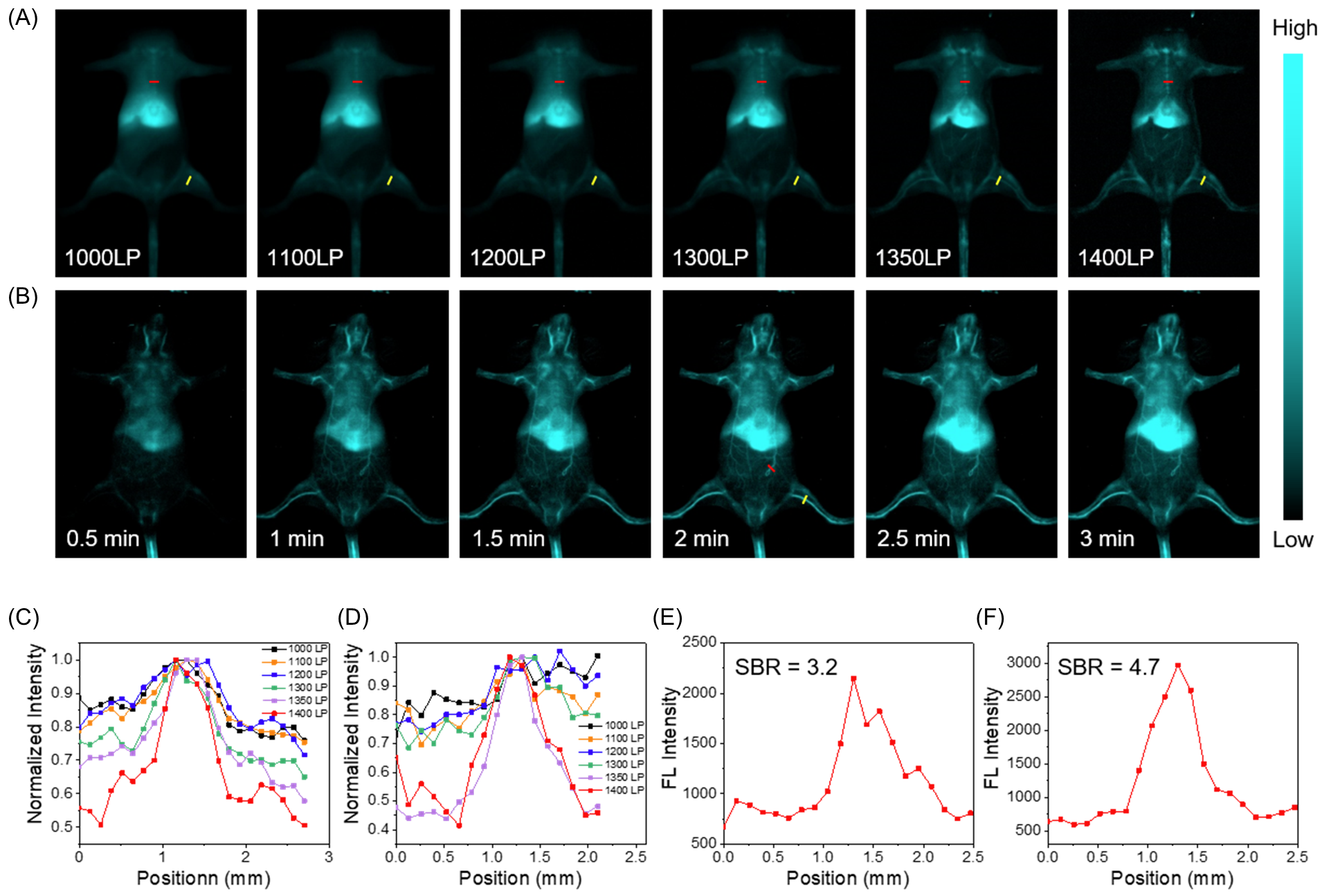


**Figure S6.** The whole-body NIR-II fluorescence imaging of BALB/c mice after tail vein injection of FC-NEt_2_. (A) The whole-body imaging after 10 min of injection with different long-pass filters. 1000 LP, 5 ms; 1100 LP, 15 ms; 1200 LP, 50 ms; 1300 LP, 300 ms; 1350 LP, 800 ms; 1400 LP, 1000 ms. Laser: 808 nm. (B) The whole-body imaging within 3 min. Laser: 808 nm; Exposure time: 800 ms; Filter: 1350 nm long-pass. Cross-sectional intensity profiles along the red line (C) and yellow line (D) in (A). Cross-sectional intensity profiles along the red line (E) and yellow line (F) in (B).

**6. Imaging and resection prostate tumor and in vivo**


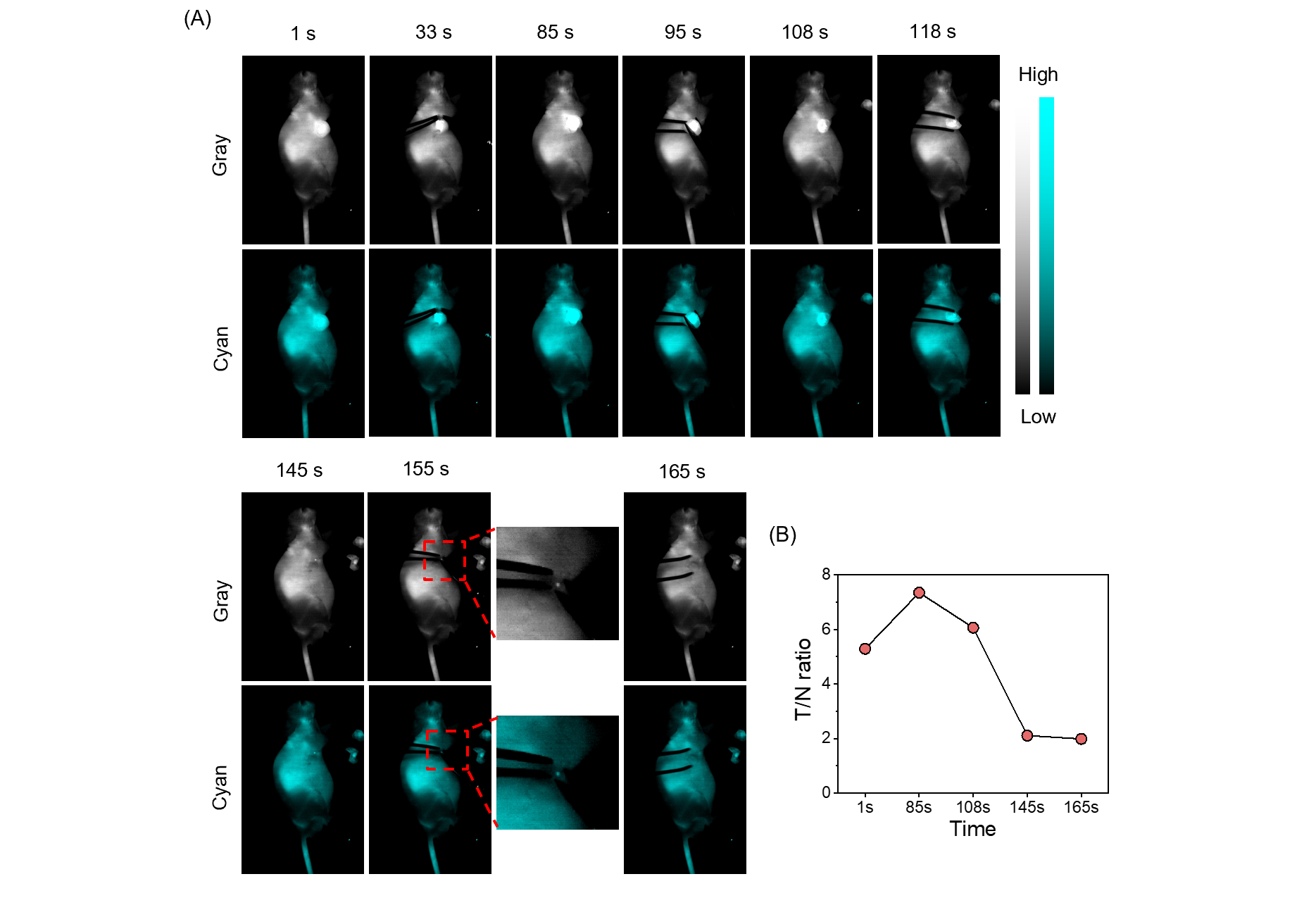


**Figure S7.** (A) NIR-II fluorescence imaging-guided tumor resection after tail vein injection of FC-PSMA for 24 h. Laser: 808 nm; Filter: 1000 nm long-pass; Exposure time: 100 ms. (B) Tumor-to-normal ratio during the surgery.

**7. Characterization of intermediate compounds, FC-NEt_2_ and FC-PSMA**


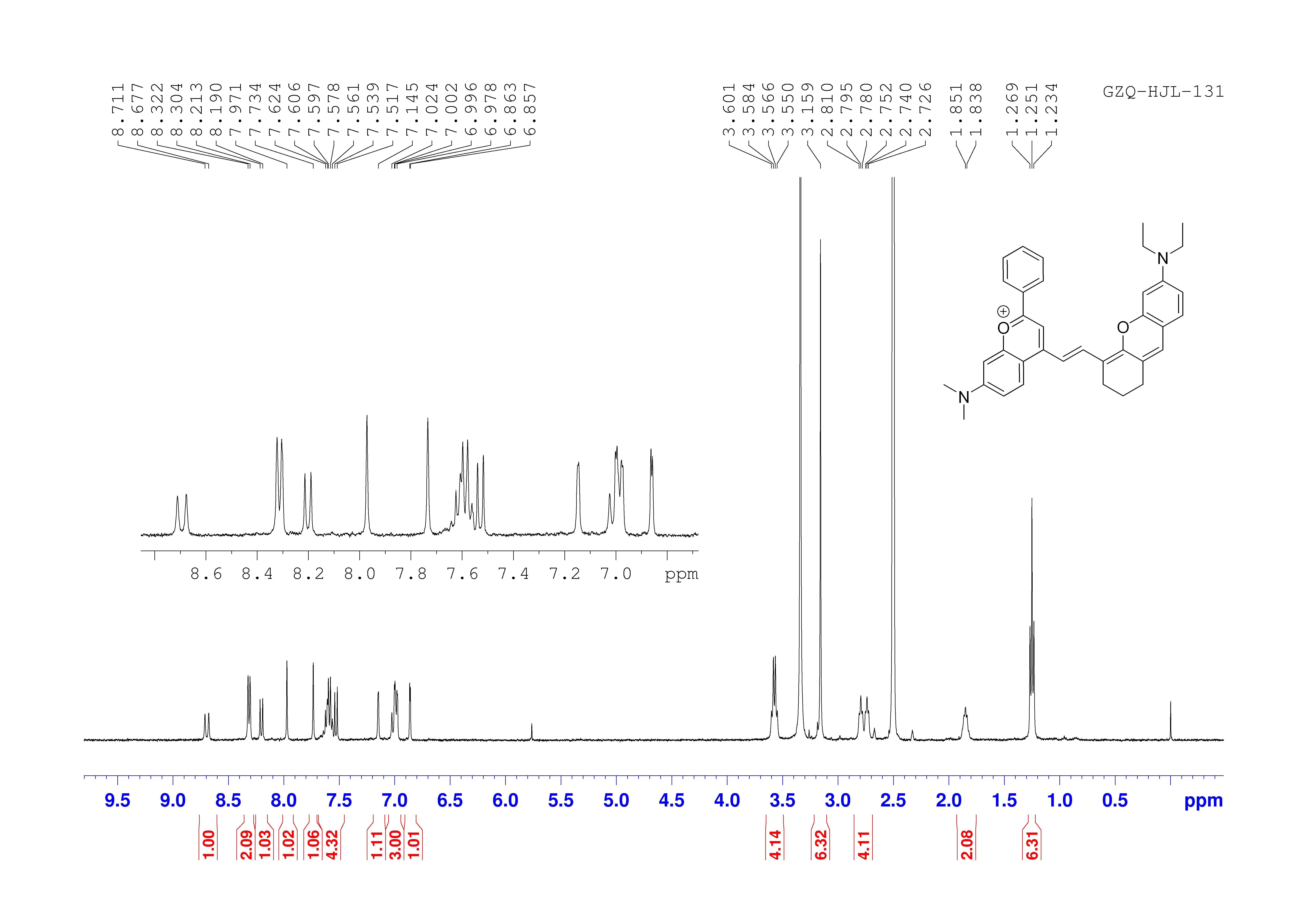


Figure S8. ^1^H NMR spectrum of **FC-NEt_2_** in DMSO-*d*_6_


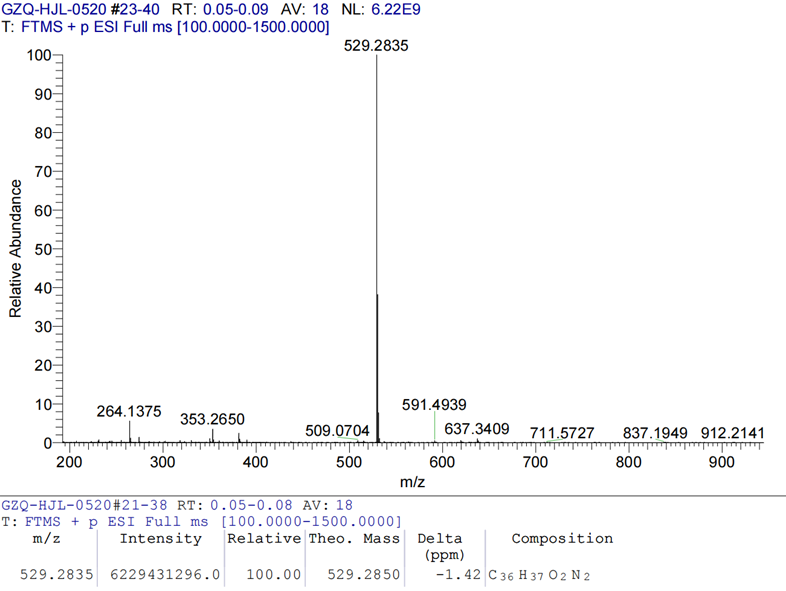


**Figure S9.** HRMS spectrum of **FC-NEt_2_**


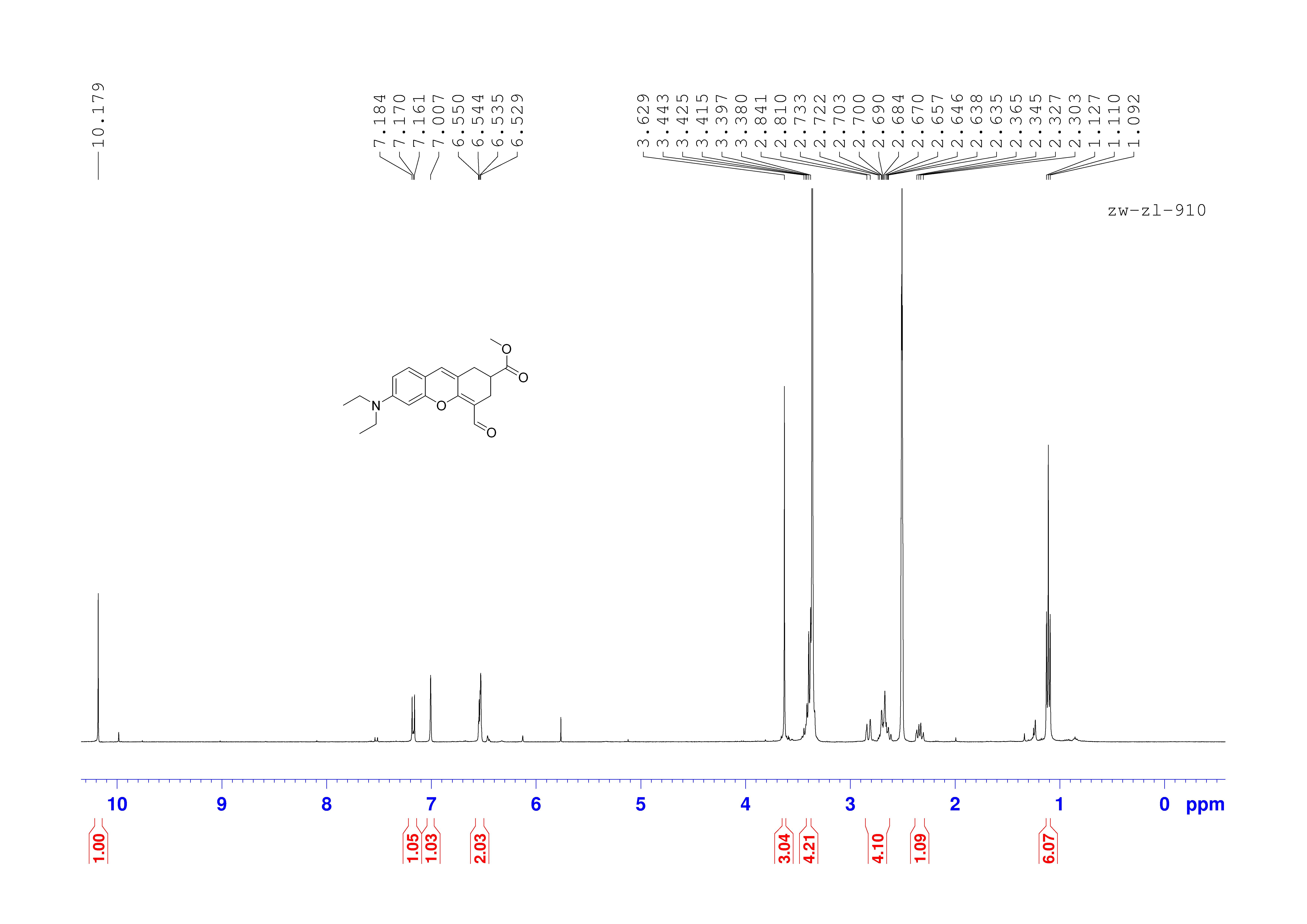


Figure S10. ^1^H NMR spectrum of **compound 2** in DMSO-*d*_6_


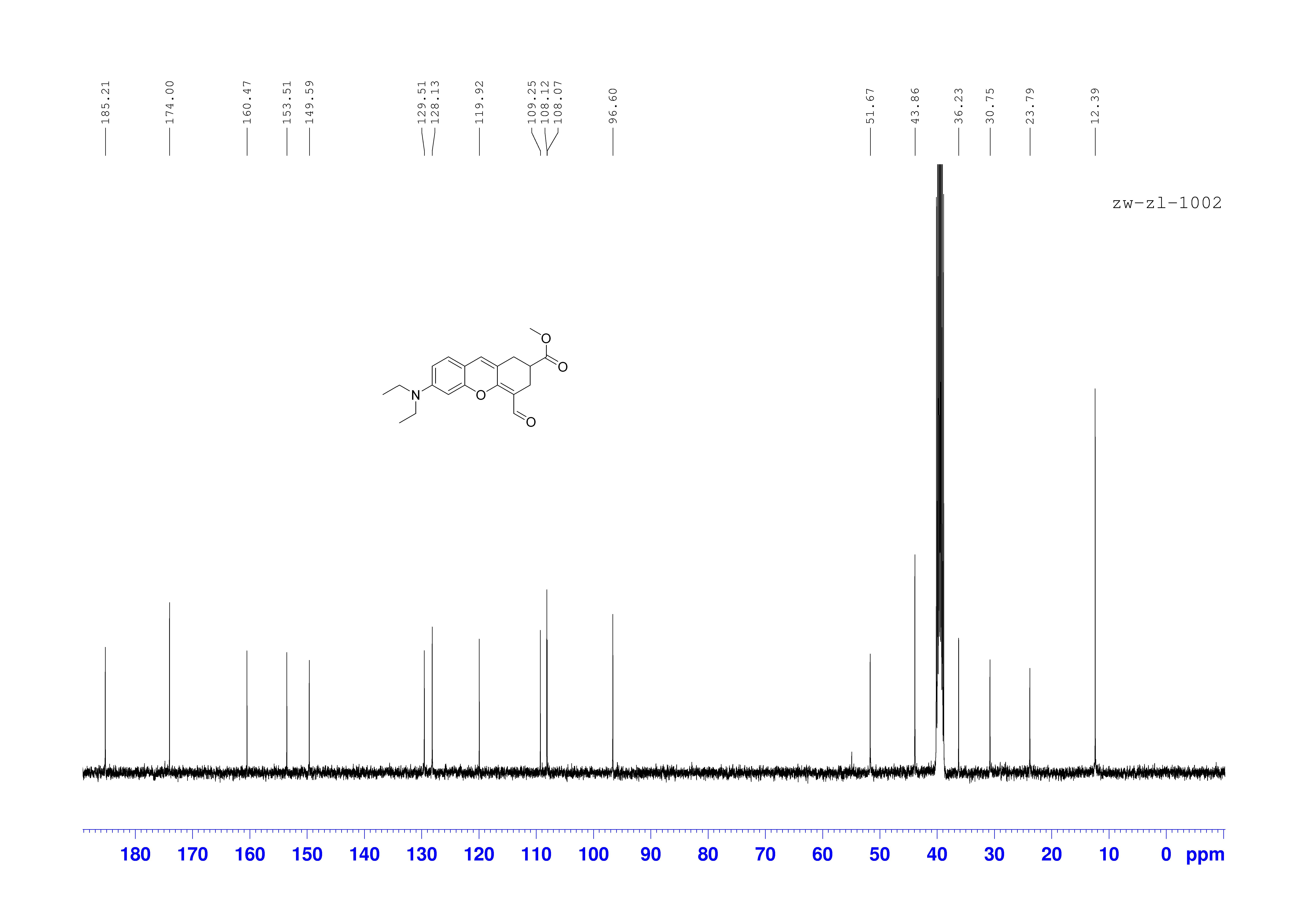


Figure S11. ^13^C NMR spectrum of **compound 2** in DMSO-*d*_6_


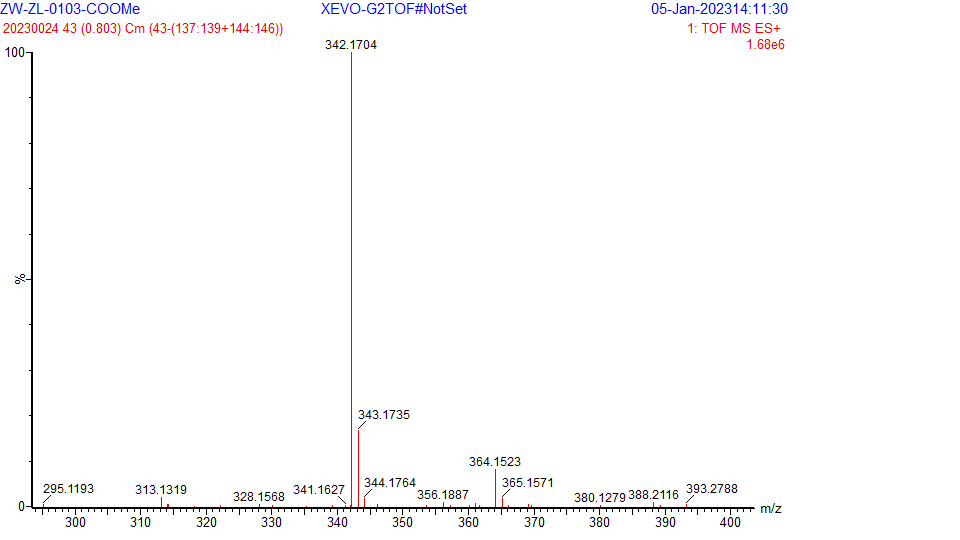


**Figure S12.** HRMS spectrum of **compound 2**


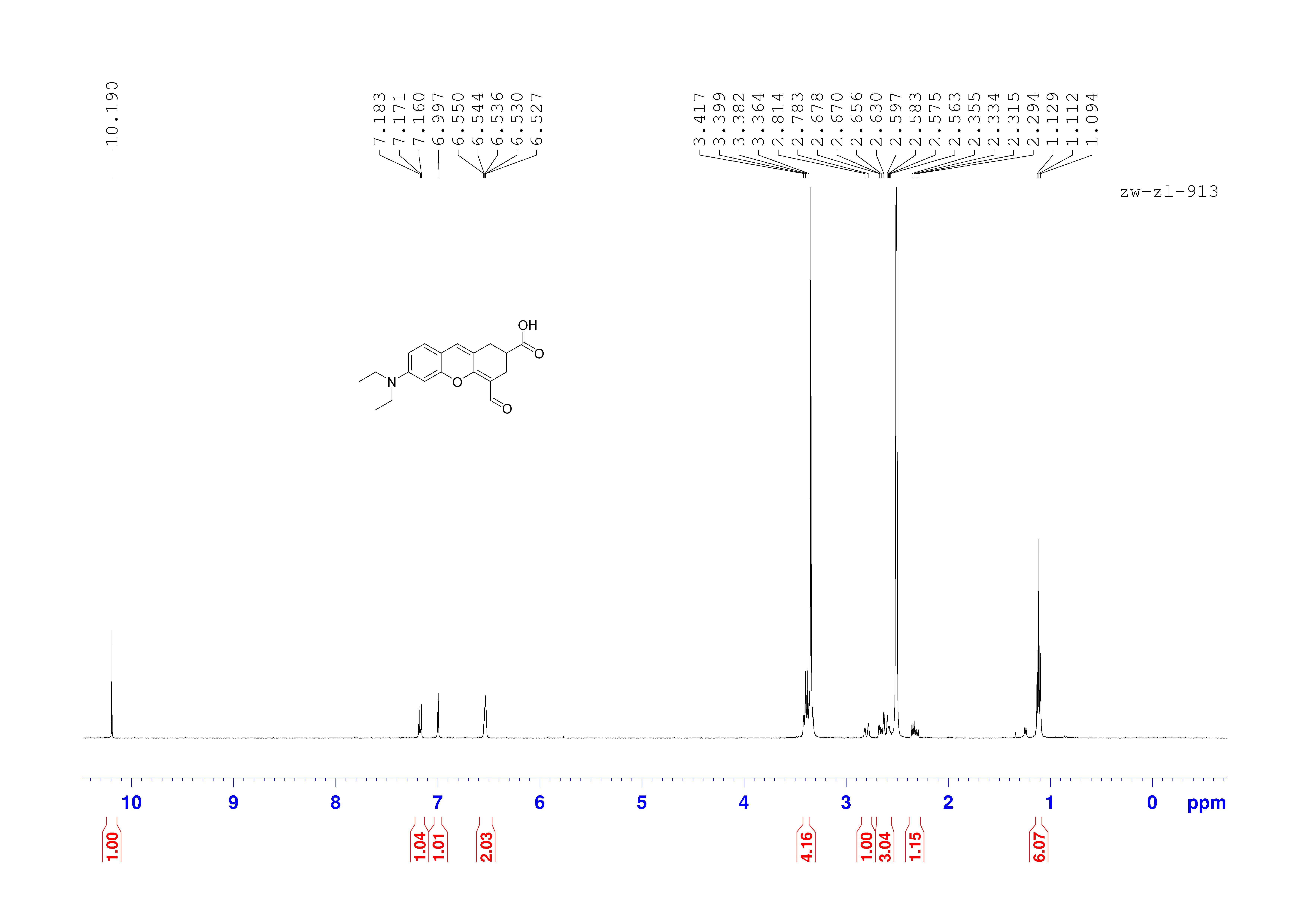


**Figure S13.** ^1^H NMR spectrum of **compound 3** in DMSO-*d*_6_


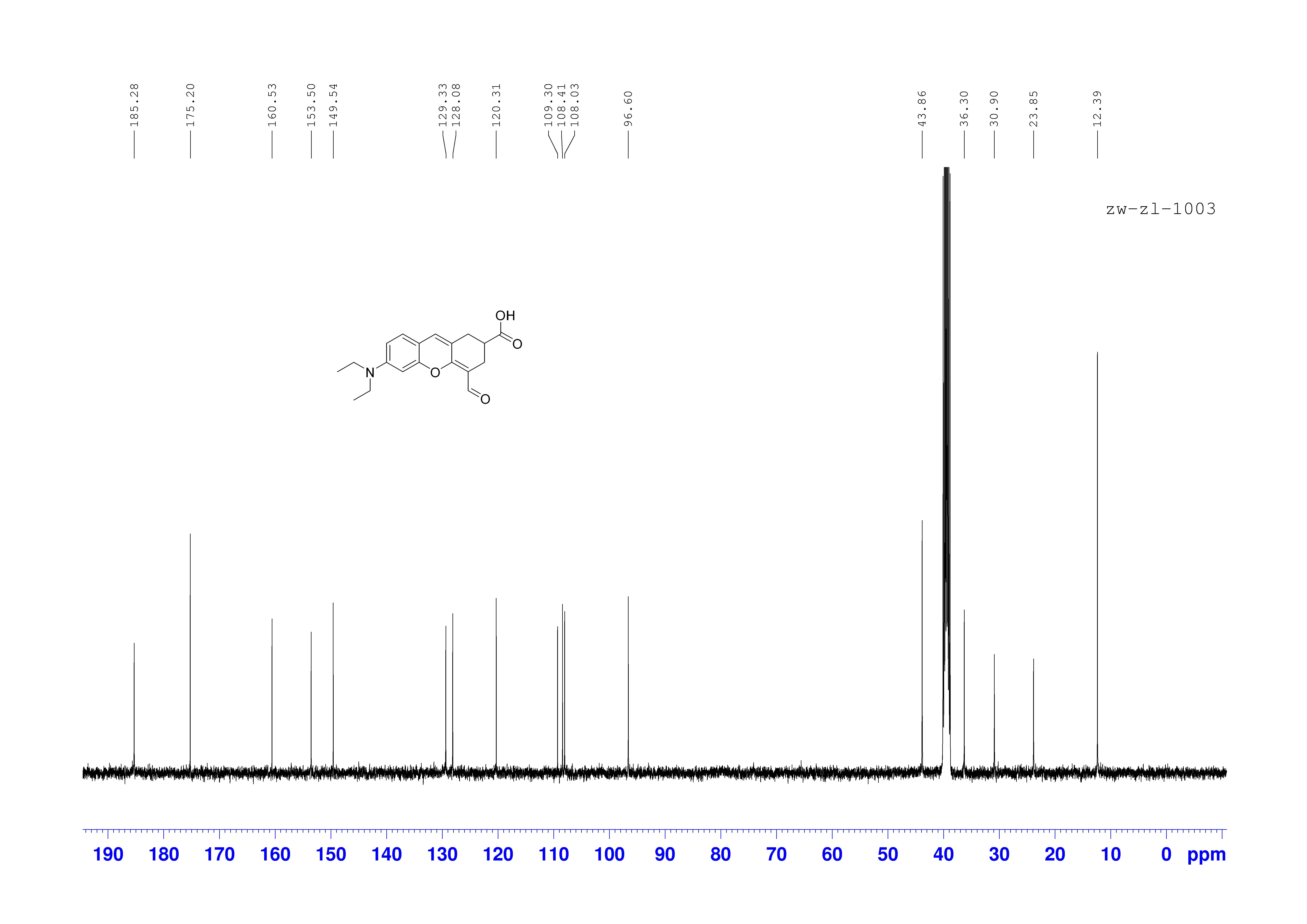


**Figure S14.** ^13^C NMR spectrum of **compound 3** in DMSO-*d*_6_


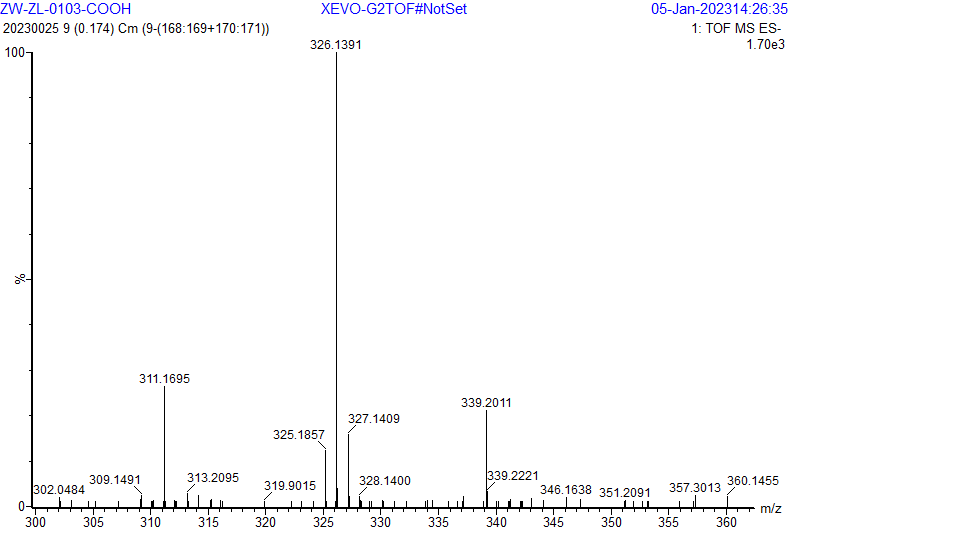


**Figure S15.** HRMS spectrum of **compound 3**


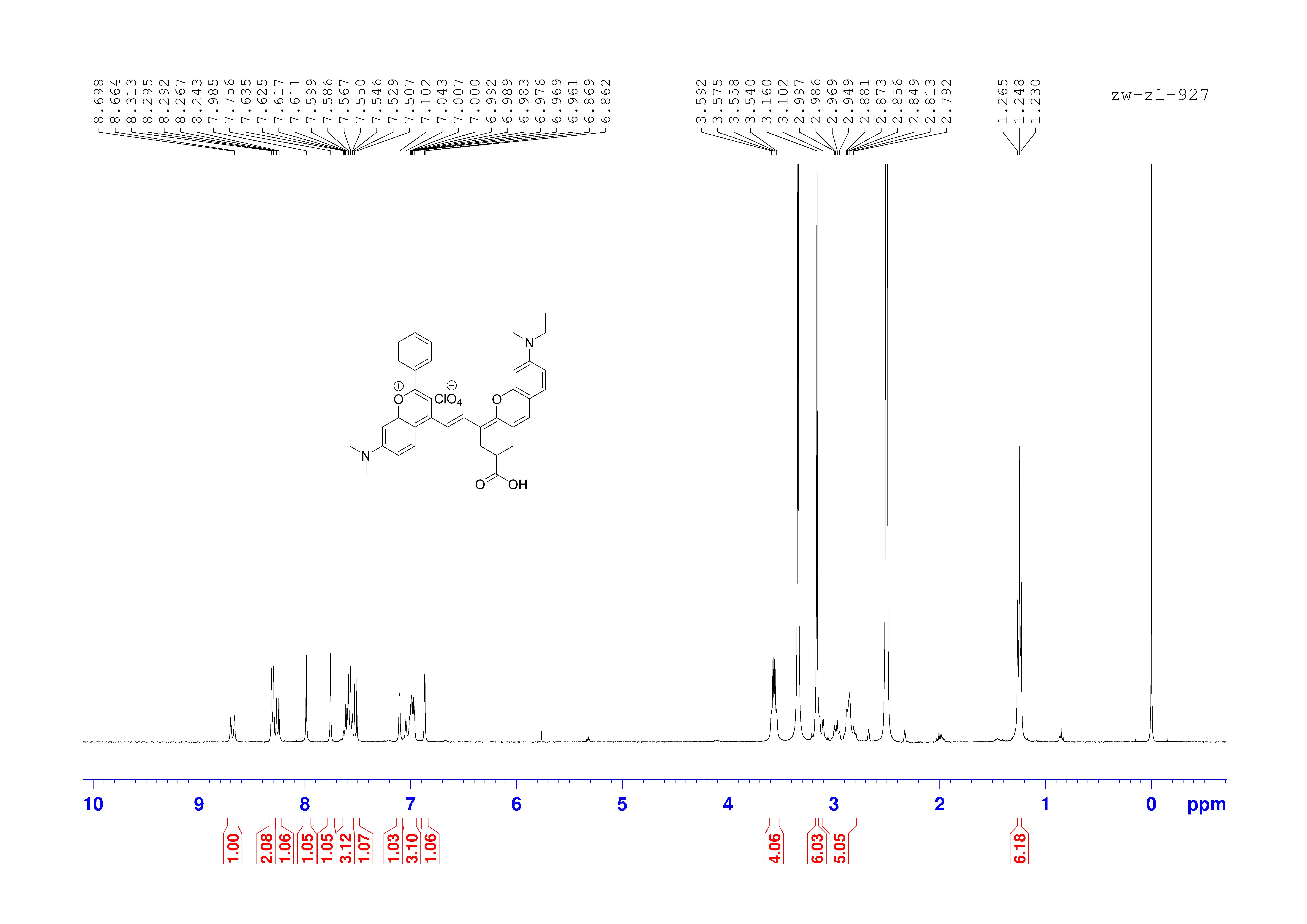


**Figure S16.** ^1^H NMR spectrum of **FC-COOH** in DMSO-*d*_6_


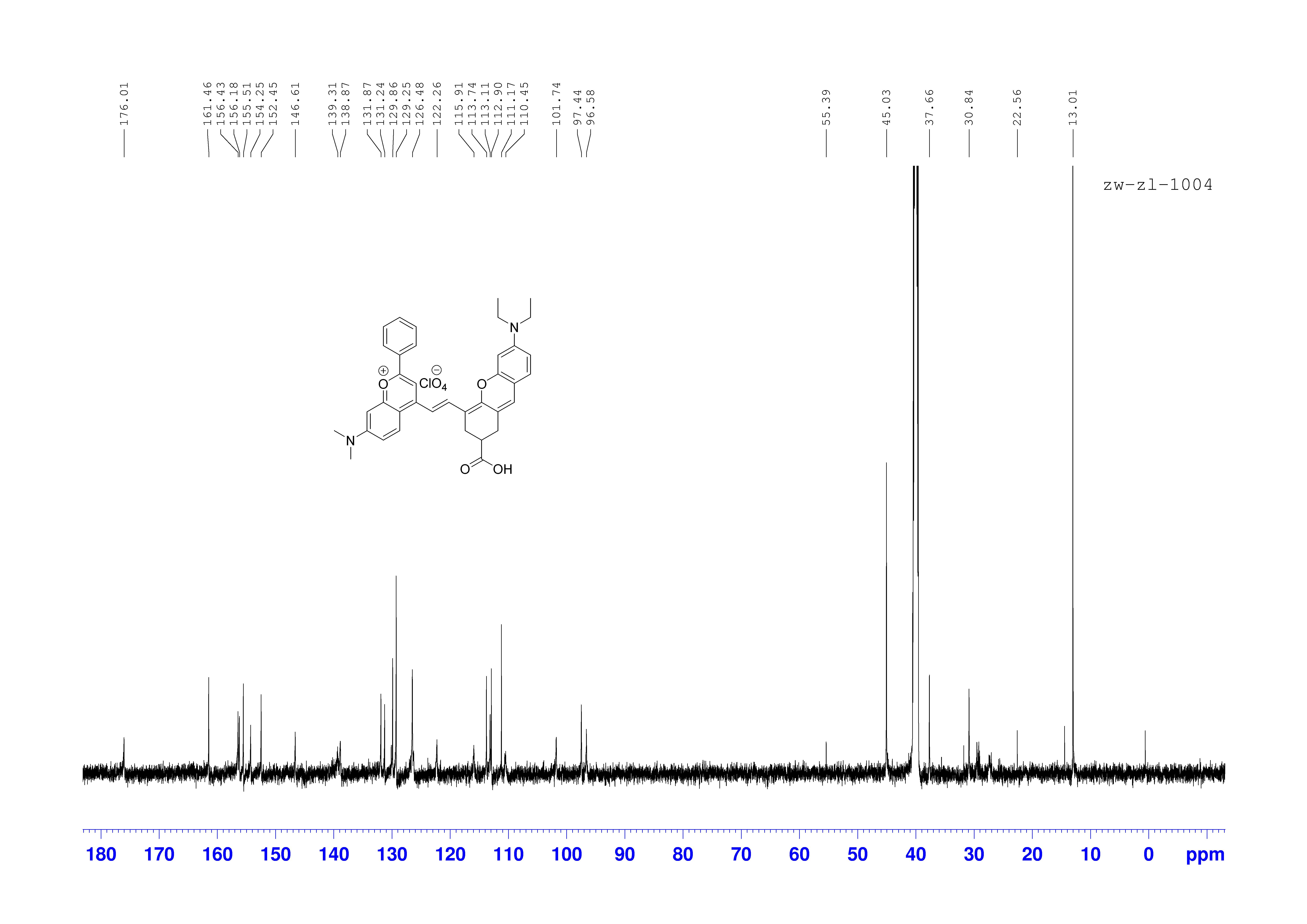


**Figure S17.** ^13^C NMR spectrum of **FC-COOH** in DMSO-*d*_6_


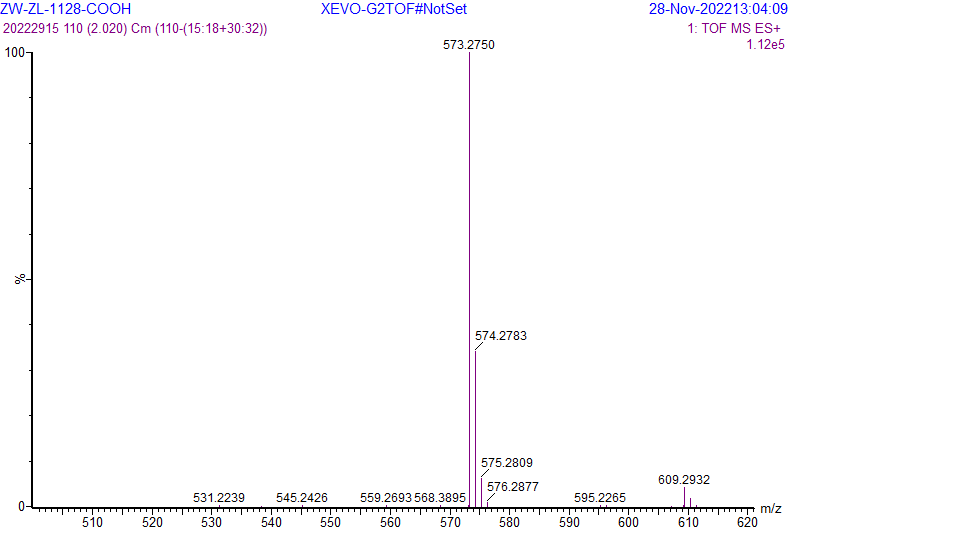


**Figure S18.** HRMS spectrum of **FC-COOH**


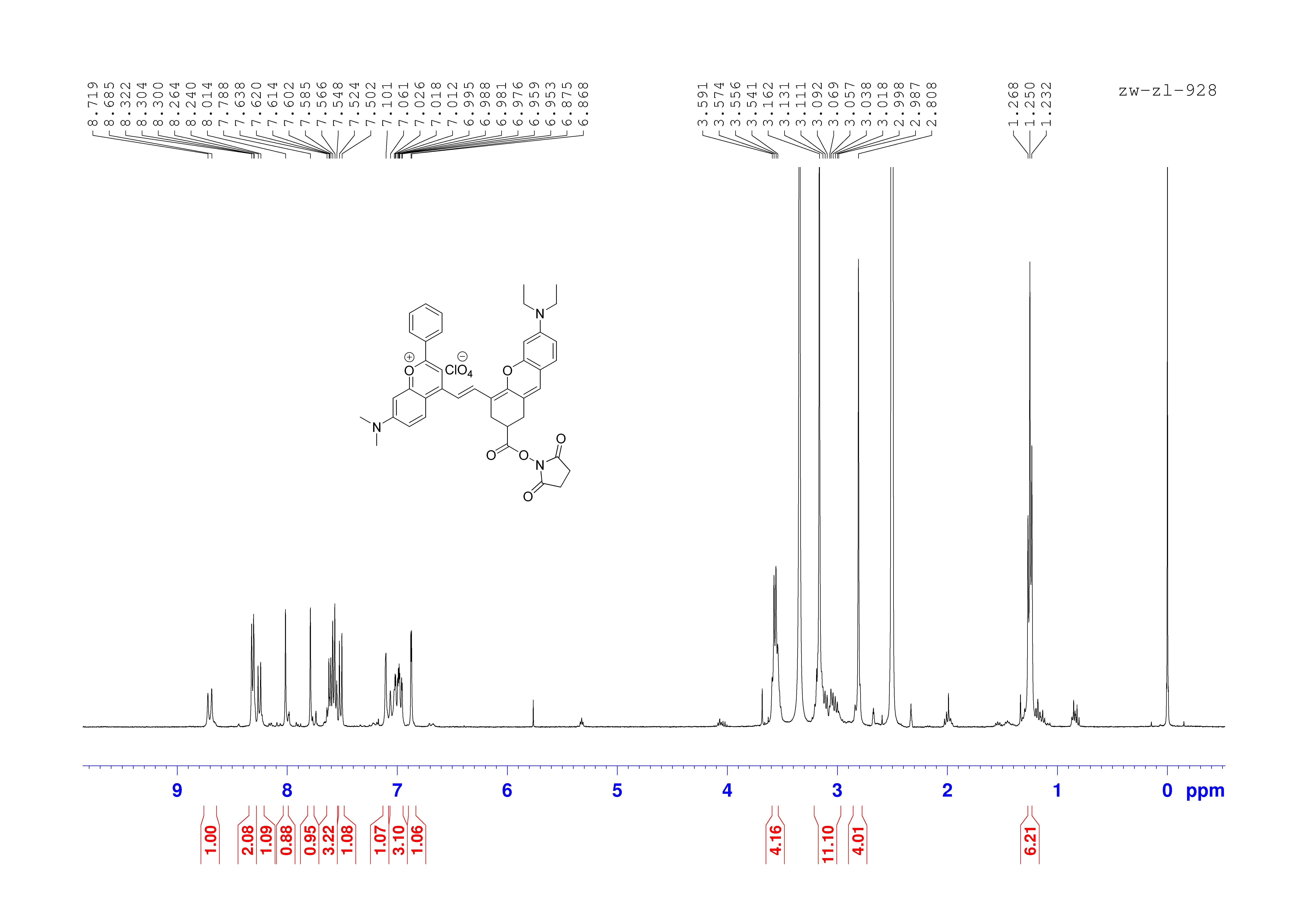


**Figure S19.** ^1^H NMR spectrum of **FC-NHS** in DMSO-*d*_6_


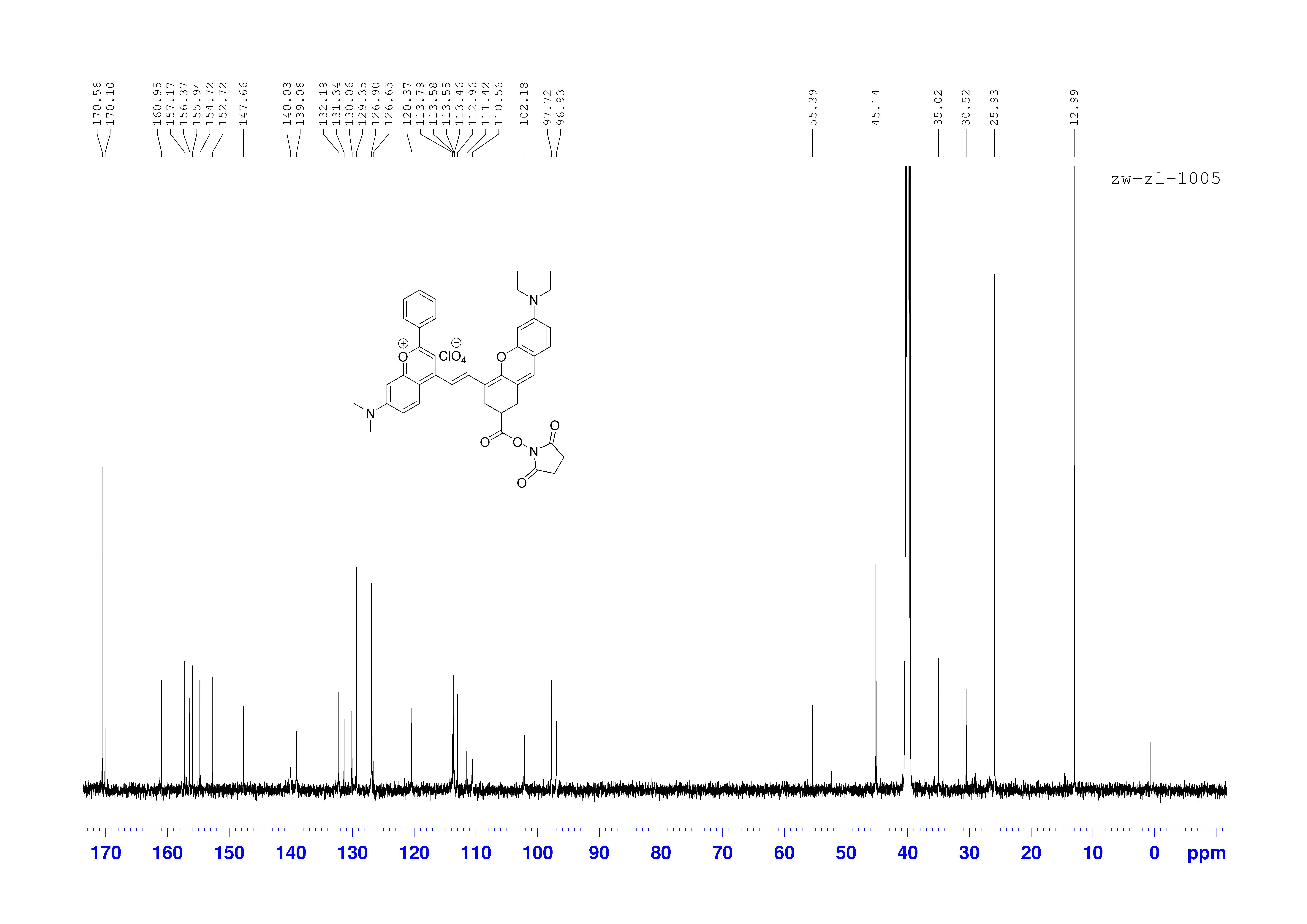


**Figure S20.** ^13^C NMR spectrum of **FC-NHS** in DMSO-*d*_6_


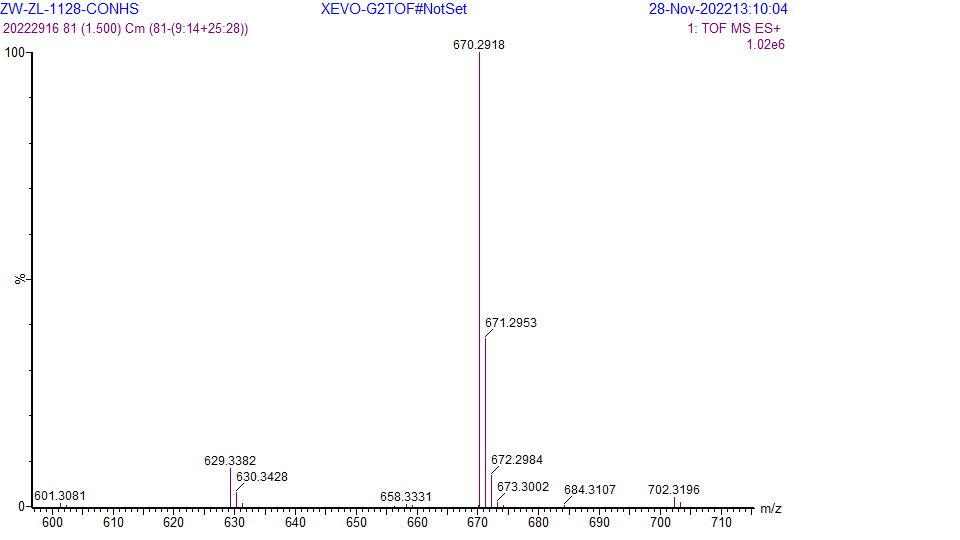


**Figure S21.** HRMS spectrum of **FC-NHS**


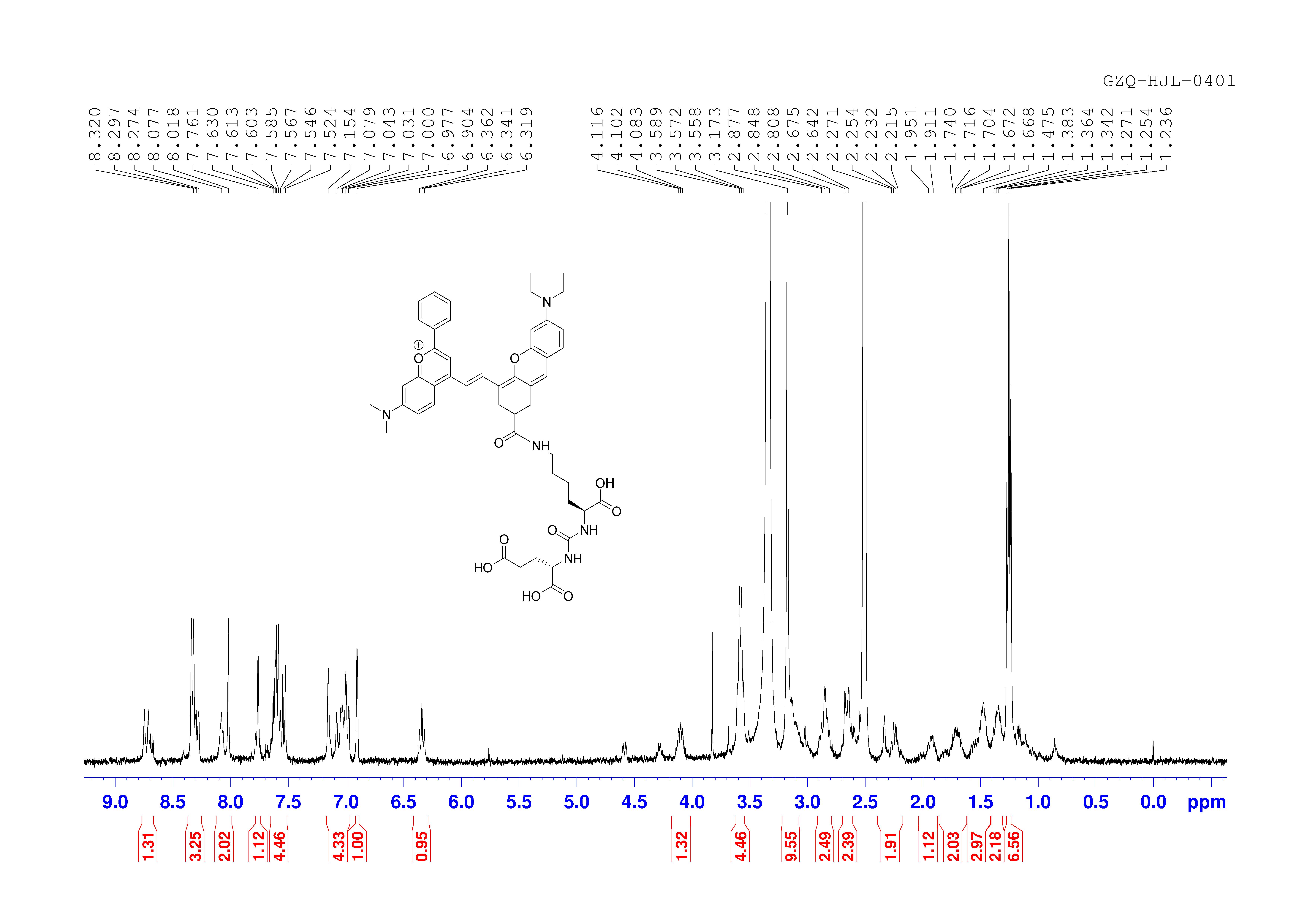


**Figure S22.** ^1^H NMR spectrum of **FC-PSMA** in DMSO-*d*_6_


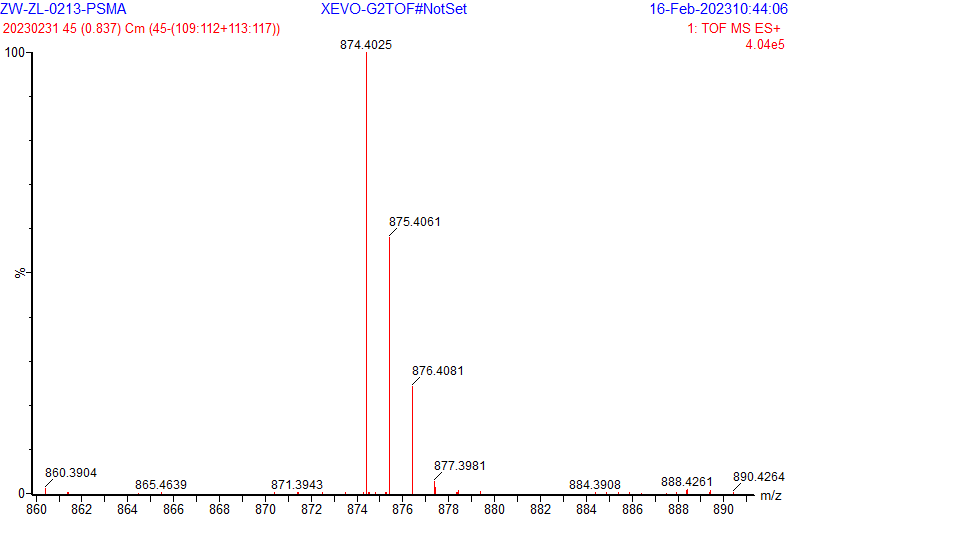


**Figure S23.** HRMS spectrum of **FC-PSMA**


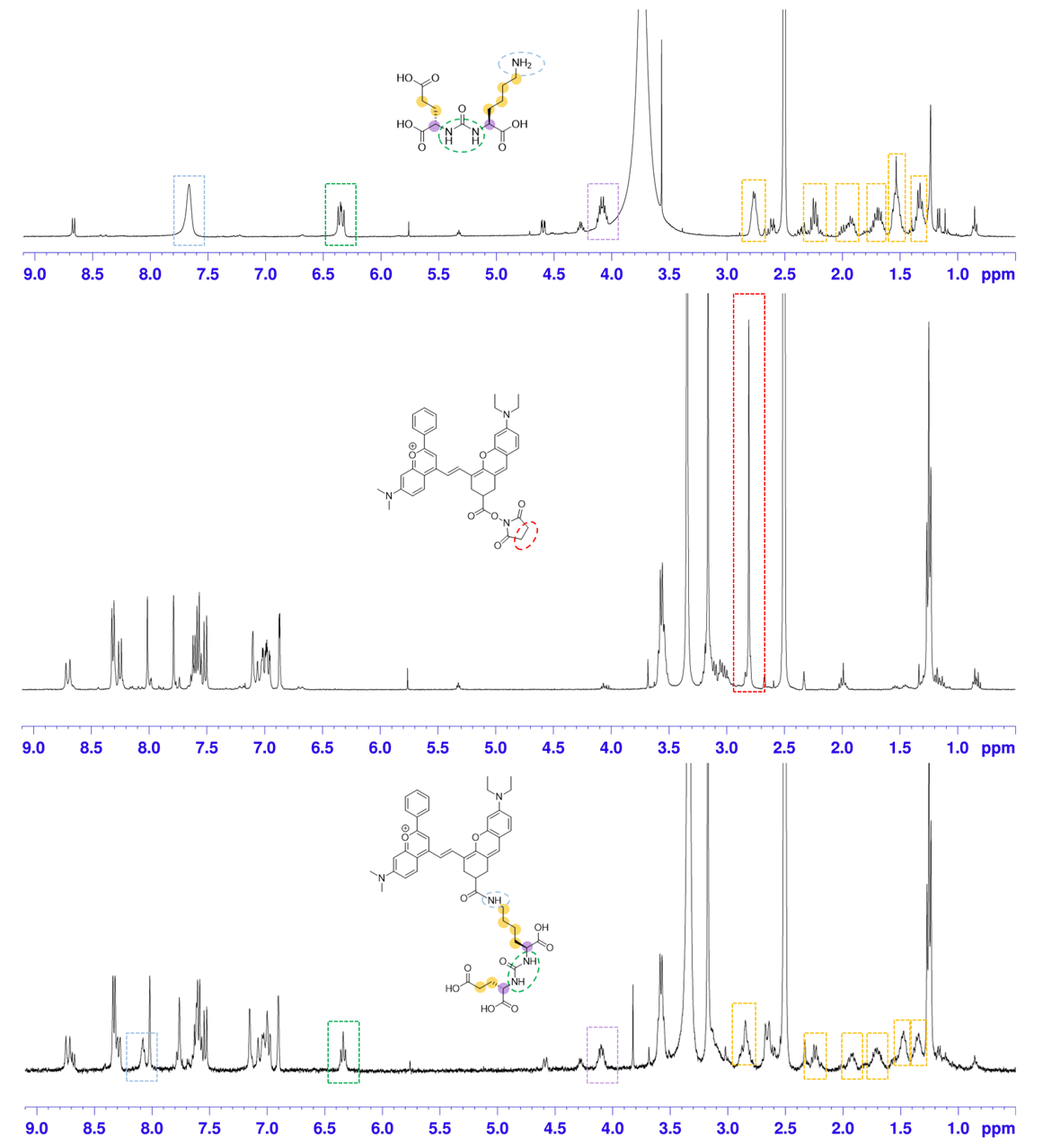


**Figure S24.** ^1^H NMR spectra of **Glu-urea-Lys**, **FC-NHS** and **FC-PSMA** in DMSO-*d*_6_

**8. References**

[1] M. J. Frisch, G. W. Trucks, H. B. Schlegel, G. E. Scuseria, M. A. Robb, J. R. Cheeseman, G. Scalmani, V. Barone, G. A. Petersson, H. Nakatsuji, X. Li, M. Caricato, A. V. Marenich, J. Bloino, B. G. Janesko, R. Gomperts, B. Mennucci, H. P. Hratchian, J. V. Ortiz, A. F. Izmaylov, J. L. Sonnenberg, Williams, F. Ding, F. Lipparini, F. Egidi, J. Goings, B. Peng, A. Petrone, T. Henderson, D. Ranasinghe, V. G. Zakrzewski, J. Gao, N. Rega, G. Zheng, W. Liang, M. Hada, M. Ehara, K. Toyota, R. Fukuda, J. Hasegawa, M. Ishida, T. Nakajima, Y. Honda, O. Kitao, H. Nakai, T. Vreven, K. Throssell, J. A. Montgomery Jr., J. E. Peralta, F. Ogliaro, M. J. Bearpark, J. J. Heyd, E. N. Brothers, K. N. Kudin, V. N. Staroverov, T. A. Keith, R. Kobayashi, J. Normand, K. Raghavachari, A. P. Rendell, J. C. Burant, S. S. Iyengar, J. Tomasi, M. Cossi, J. M. Millam, M. Klene, C. Adamo, R. Cammi, J. W. Ochterski, R. L. Martin, K. Morokuma, O. Farkas, J. B. Foresman and D. J. Fox, Journal, **2016**.

[2] F. Weigend, R. Ahlrichs, Phys, *Chem. Chem. Phys.* **2005**, 7, 3297-3305

[3] A. V. Marenich, C. J. Cramer, D. G. Truhlar, J. *Phys. Chem. B* **2009**, 113, 6378-6396.

[4] T. Lu, F. Chen, *J. Comput. Chem.* **2012**, 33, 580-592.

[5] E. D. Cosco, J. R. Caram, O. T. Bruns, D. Franke, R. A. Day, E. P. Farr, M. G. Bawendi, E. M. Sletten, *Angew. Chem., Int. Ed.* **2017**, *56*, 13126-13129.

[6] A. Romieu, J.-A. Richard, *Tetrahedron Lett.* **2016**, *57*, 317-320.

[7] Z. Zhao, Y. Zhang, M. Wu, C. Yan, Z. Guo, *Chem. Biomed. Imaging* **2023**, *1*, 620-627.

[8] L. Zhang, Y. Zhang, W. Chi, C. Yan, Z. Zhao, X. Liu, W.-H. Zhu, Z. Guo, *ACS Mater. Lett.* **2022**, *4*, 1493-1502.
