# Supplementary figures and images for: “Crossbreeding” NIR‐II flavchromene for PSMA‐positive prostate cancer detection and image‐guided surgery
Source: Smart Mol. 2024 Jul 4;2(3):e20240020. doi: 10.1002/smo.20240020 (PMC12118234; doi:10.1002/smo.20240020)

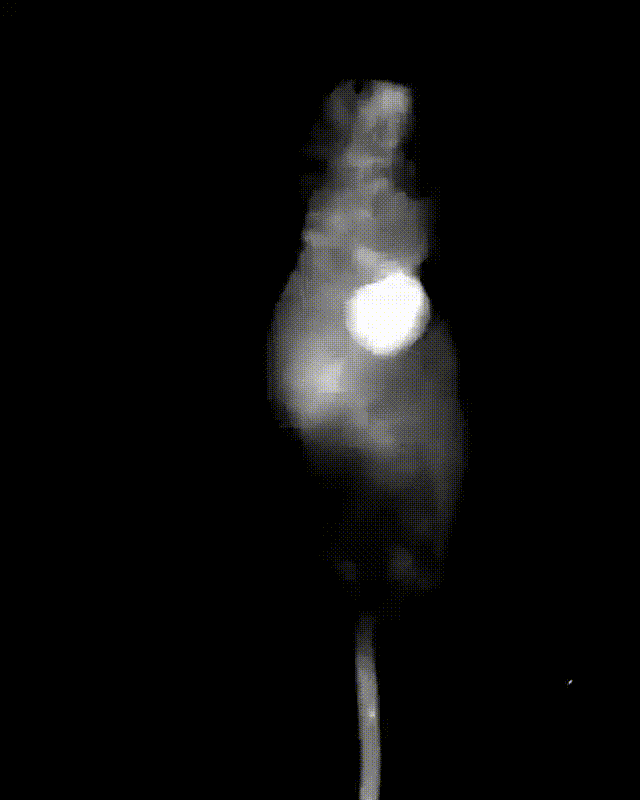

Supplement: Supplementary file 2 — Video S1 [file SMO2-2-e20240020-s002.gif]
